# Disruption of redox homeostasis for combinatorial drug efficacy in *K-Ras* tumors as revealed by metabolic connectivity profiling

**DOI:** 10.1186/s40170-020-00227-4

**Published:** 2020-09-29

**Authors:** Daniela Gaglio, Marcella Bonanomi, Silvia Valtorta, Rohit Bharat, Marilena Ripamonti, Federica Conte, Giulia Fiscon, Nicole Righi, Elisabetta Napodano, Federico Papa, Isabella Raccagni, Seth J. Parker, Ingrid Cifola, Tania Camboni, Paola Paci, Anna Maria Colangelo, Marco Vanoni, Christian M. Metallo, Rosa Maria Moresco, Lilia Alberghina

**Affiliations:** 1 Institute of Molecular Bioimaging and Physiology (IBFM), National Research Council (CNR), Segrate, MI Italy; 2 ISBE. IT/Centre of Systems Biology, Piazza della Scienza 4, 20126 Milan, Italy; 3 Department of Biotechnology and Biosciences, University of Milano-Bicocca, Piazza della Scienza 2, 20126 Milan, Italy; 4 Department of Medicine and Surgery and Tecnomed Foundation, University of Milano-Bicocca, Via Cadore 48, 20900 Monza, Italy; 5 Institute for Systems Analysis and Computer Science “Antonio Ruberti”, National Research Council, Rome, Italy; 6 Nuclear Medicine Unit, IRCCS San Raffaele Scientific Institute, Milan, Italy; 7 Department of Bioengineering, University of California, San Diego, La Jolla, CA USA; 8 Moores Cancer Center, University of California, San Diego, La Jolla, CA USA; 9 Institute for Biomedical Technologies (ITB), National Research Council (CNR), Segrate, Milan, Italy; 10 Department of Computer, Control and Management Engineering, Sapienza University of Rome, Rome, Italy

**Keywords:** Metabolic rewiring, Metabolic cancer therapy, Metabolic signature, Glycolysis, Glutamine, Combinatorial drug treatment, Precision oncology, Metabolic connectivity

## Abstract

**Abstract:**

**Background:**

Rewiring of metabolism induced by oncogenic *K-Ras* in cancer cells involves both glucose and glutamine utilization sustaining enhanced, unrestricted growth. The development of effective anti-cancer treatments targeting metabolism may be facilitated by the identification and rational combinatorial targeting of metabolic pathways.

**Methods:**

We performed mass spectrometric metabolomics analysis in vitro and in vivo experiments to evaluate the efficacy of drugs and identify metabolic connectivity.

**Results:**

We show that *K-Ras*-mutant lung and colon cancer cells exhibit a distinct metabolic rewiring, the latter being more dependent on respiration. Combined treatment with the glutaminase inhibitor CB-839 and the PI3K/aldolase inhibitor NVP-BKM120 more consistently reduces cell growth of tumor xenografts. Maximal growth inhibition correlates with the disruption of redox homeostasis, involving loss of reduced glutathione regeneration, redox cofactors, and a decreased connectivity among metabolites primarily involved in nucleic acid metabolism.

**Conclusions:**

Our findings open the way to develop metabolic connectivity profiling as a tool for a selective strategy of combined drug repositioning in precision oncology.

## Background

A metabolic rewiring in which glucose is converted to lactate, while glutamine-derived α-ketoglutarate (Akg) enters the tricarboxylic acid cycle (TCA)—undergoing partial reductive carboxylation to citrate [1]—characterize a large number of cancer cells. This basic scheme may be modified by the presence in the cellular environment (both in vitro and in vivo) of lactate and/or amino acids such as proline, arginine, and asparagine, thereby generating a variety of complex and flexible metabolic pathways sustaining the enhanced and unrestricted growth of cancer cells [2–5].

Genetic [6, 7] and tissue-related factors, as well as nutrients and cytokines in the stromal environment of the tumor, affect cancer metabolic rewiring (CMR) [8, 9], i.e., CMR may be activated by oncogenic *K-Ras*, which is mutated in approximately 35% of lung adenocarcinomas and 45% of colorectal cancers. *K-Ras* activation increases tumorigenicity, promotes environmental adaptation and acquisition of drug resistance, resulting in poor prognosis [9].

Although metabolic rewiring has already been proposed to open therapeutic windows for new drugs, the connectivity and heterogeneity of cancer metabolism have not allowed full exploitation of CMR for precision oncology yet [10]. The high degree of connectivity in metabolism requires integrating experimental metabolic profiling and innovative computational-network analysis to extract more in-depth information. This new approach will open the way to systems metabolomics as a new, robust, customizable tool for cancer precision medicine [11].

The CMR inhibitor CB-839 is a reversible non-competitive allosteric glutaminase (GLS) inhibitor, exhibiting anti-proliferative activity in triple-negative breast cancer cell lines, and in xenografts [12]. CB-839 is well tolerated in pre-clinical studies (no weight loss or toxicity in mice) but shows reduced in vivo activity compared with in vitro studies [1, 13]. On the other hand, inhibitors of signaling pathways, such as the pan-PI3K inhibitor NVP-BKM120 (BKM120), are known to inhibit cell growth and decrease glucose consumption by decreasing the release of active aldolase from the actin cytoskeleton both in vitro and in vivo [14].

To gain a more in-depth insight into the interplay between oncogenic *K-Ras*, tissue origin, cancer metabolic rewiring, and drug sensitivity, here, we provide evidence that *K-Ras*^G12S^ A549 lung cancer and *K-Ras*^G13D^ HCT116 colon cancer cells present substantial differences in their metabolic rewiring: as a result, the latter cell line appears to be more dependent on respiration. Combinatorial drug treatment with the glutaminase inhibitor CB-839 and the PI3K/aldolase inhibitor BKM120 effectively down-regulates the growth of A549 and HCT116 xenografts. The double treatment disrupts redox homeostasis and dramatically reduces the inter-pathway connectivity of metabolites involved primarily in nucleic acid metabolism. Since metabolic profiling and its connectivity analysis effectively recapitulate information obtainable from more extensive analyses, including metabolic pathway identification by isotope labeling and metabolic flux analysis, we propose using metabolic connectivity profiling as a tool for selecting more effective combined drug treatments in precision oncology.

## Methods

### Cell culture and mice

Cell culture, cell proliferation analysis, cell treatments, and mice are described in detail in the Supplementary Materials and Methods.

### Glucose uptake, lactate production, and seahorse metabolic analysis

Glucose and glutamine uptake and lactate and glutamate secretion YSI analysis, Seahorse XF Cell Mito Stress Test, Aldolase activity in cell samples, ROS levels measurement, ATP quantification in cell samples, ALT and AST activity in liver tissues, and NADP/NADPH assay were detailed described in the Supplementary Materials and Methods.

### Metabolites extraction and metabolic profiling

Metabolites extraction from cell culture and tissue samples detailed protocol and GC-MS and LC-MS detailed information are described in the Supplementary Materials and Methods.

### 13-C Metabolic flux analysis

13C MFA was carried out using INCA v1.7 based on Elementary Metabolite Unit (EMU) framework [15, 16]. Flux through a metabolic network consisting of Glycolysis, PPP, TCA, FA, & Biomass synthesis was constructed [17] and was estimated by the least-squares regression of metabolite labeling pattern and measured extracellular fluxes. The network’s flux values were iteratively adjusted using a Levenberg-Marquardt (local search) algorithm to minimize the sum of squared residual (SSR) objective function. The best global fit was found after estimating at least 50 times using random initial guesses for all reactions in the metabolic network. All the fluxes were subjected to chi-square statistical test to assess goodness of fit, and 95% confidence intervals were computed [18]. Schematic representation of fluxes was performed using the Escher software https://escher.github.io/#/.

### Animal model, positron emission tomography imaging, and pharmacological therapy

Animal models, positron emission tomography imaging and PET imaging and quantification detailed are described in the Supplementary Materials and Methods.

NVP-BKM120 was formulated in NMP/PEG300 (10/90, v/v). The solution was freshly prepared daily just before gavaging by dissolving the powder, first in N-Methyl-2-pyrrolidone (NMP, Sigma Aldrich) with sonication and then by adding the remaining volume of PEG300 (Sigma Aldrich) as previously described [19]. The application volume was 10 mL/kg. CB-839 was dissolved in a vehicle containing 25% (w/v) hydroxypropyl-b-cyclodextrin (Cayman Chemical Company) in 10 mmol/L citrate (pH 2.0). The formulation was 20 mg/mL for a final dosing volume of 10 mL/kg, as previously described [12]. For labeling experiments, 1 M [U-13C6] glucose in sterile PBS was infused injecting 80 μl (20 mg) of solution at 15-min intervals three times before the sacrifice. For survival study, treatments were administered until (1) a tumor dimension reached 15 mm, (2) both dimensions exceeded 10 mm, or (3) for evident signs of disease (i.e., motion difficulty). To calculate tumor growth inhibition, we used the following formula: TGI = [1 − (TF/T0)A/(TF/T0)V] × 100, where TF is the time point analyzed, T0 is the initial time, A is the corresponding drug, and V is the vehicle [20].

### Quantification and statistical analysis

Results are expressed as mean value ± SD. Experimental differences were tested for significance with the Student’s *t* test or, when possible, with the two-way ANOVA test. A *p* value of 0.05 or less was considered statistically significant. Statistics are included in the figure legends.

### Connectivity pattern correlation

Connectivity heatmaps were obtained by calculating Spearman correlation coefficients among each pair of metabolic traits and cell number (or volume) for A549 and HCT116 tumor cells (tissues) in the control condition and under single or combined drug treatments.

### Pathway connectivity analysis

To summarize the overall profile of a given pathway, metabolites were assigned to different pathways using KEGG. We computed the pathway “eigenmetabolite” defined as the first principal component of the metabolites belonging to each pathway [21]. Thus, the pathway eigenmetabolite can be considered a representative metabolite able to condense each pathway into one and can be used to explore the relationships among the different pathways in control and treatment conditions (Figure S6 S5C).

### Connectivity network

In metabolic networks of connectivity, nodes represent metabolites and a link occurs between two nodes if the absolute value of Spearman correlation between their expression levels is greater than a selected threshold (i.e., 0.6) and statistically significant (FDR < 0.05). Modules in the network correspond to locally dense subgraphs obtained by using the Cytoscape plugin MCODE.

We visualized the screened cluster networks with Cytoscape software [22] and identified the most significant clusters using the Cytoscape plugin MCODE (Molecular Complex Detection Algorithm) [23]. MCODE is a relatively fast method for clustering, which allows detecting highly interconnected (locally dense) regions within a network. In particular, MCODE exploits a vertex-weighting scheme based on the clustering coefficient, which measures the density of the neighborhood of a vertex. The algorithm starts from the weighted vertex networks. It seeds a module with the highest weighted vertex (node) and recursively moves outward from the seed vertex to isolate the densest regions (modules) according to given parameters. These include degree cutoff (controlling the minimum degree necessary for scoring a node), node score cutoff (controlling cluster expansion by adding only nodes with a score deviating from the seed node’s score by less than the set cutoff as new cluster members), k-core (filtering out clusters that do not contain a maximally inter-connected sub-cluster of at least k degrees), and max depth (limiting the distance from the seed node within which MCODE can search for cluster members). In our analysis, we set the MCODE parameters as follows: degree cutoff = 2, node score cutoff = 0.2, k-core = 2, max depth = 100.

Resulting modules are then ranked based on a score defined as the product of the module subnetwork density (i.e., D = |E|/|E|max, with |E| the number of module edges and |E|max the total number of the network edges) and the number of vertices in the module subnetwork. Larger, denser modules appear higher in the ranked list.

### Circos plots analysis

CIRCOS plots [24] were constructed using table viewer (http://mkweb.bcgsc.ca/tableviewer/). Up-regulated metabolites and pathways identified using Metaboanalyst [25] are shown outside of the circle.

### Transcriptome sequencing (RNA-seq) and data analysis

Transcriptome sequencing (RNA-seq) experiments and data analysis are described in the Supplementary Materials and Methods.

## Results

### Human HCT116 colon cancer cells are more respiratory than A549 lung cancer cells

**Fig. 1:**
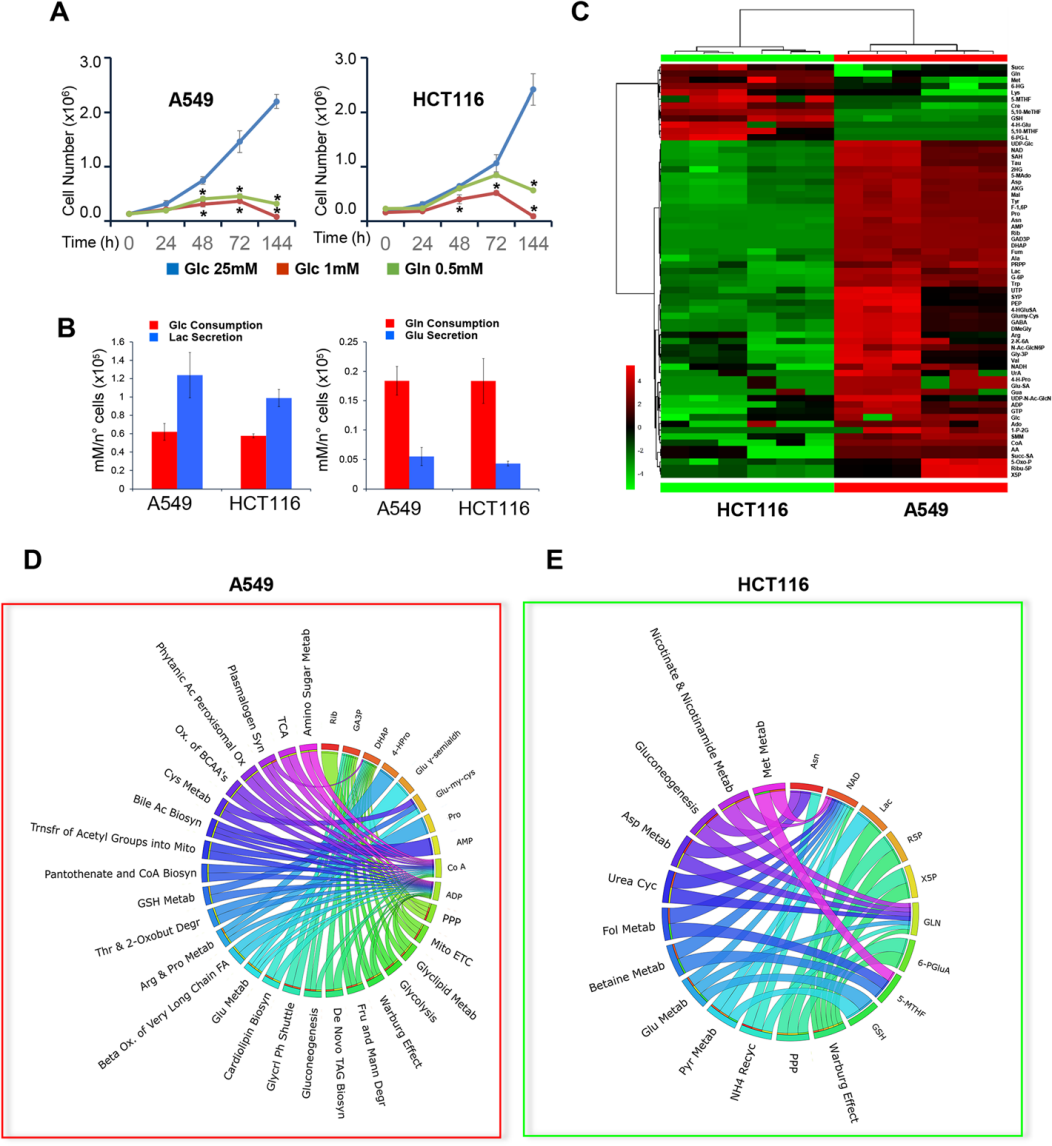
Metabolic phenotyping of human *K-Ras*^G12S^ A549 (A549) and *K-Ras*^G13D^ HCT116 (HCT116) cancer cell lines. **a** Proliferation curves of A549 and HCT116 cancer cells. Cells were plated onto 6-well plates in standard medium. The culture medium was replaced after 18 h with standard medium ( , Glc 25 mM), or medium containing 1 mM glucose ( ), or 0.5 mM glutamine ( ). Cells were collected and counted at the indicated time points. Error bars indicate SD (*n* = 3). **b** Extracellular uptake ( ) of Glc/Gln and secretion ( ) of Lac/Glu in lung and colon cancer cells grown for 48 h. **c** Untargeted metabolic profiling of A549 lung and HCT116 colorectal cancer cell lines grown in standard growth condition. Hierarchical clustering heatmaps show significantly different intracellular metabolites by LC-MS and GC-MS. **d**, **e** Circos plots show the most significant up-regulated metabolites and cognate enriched pathways in A549 cells (panel **c** **d**) and HCT116 (panel **d e**). (*p* ≤ 0.05)

**Fig. 2:**
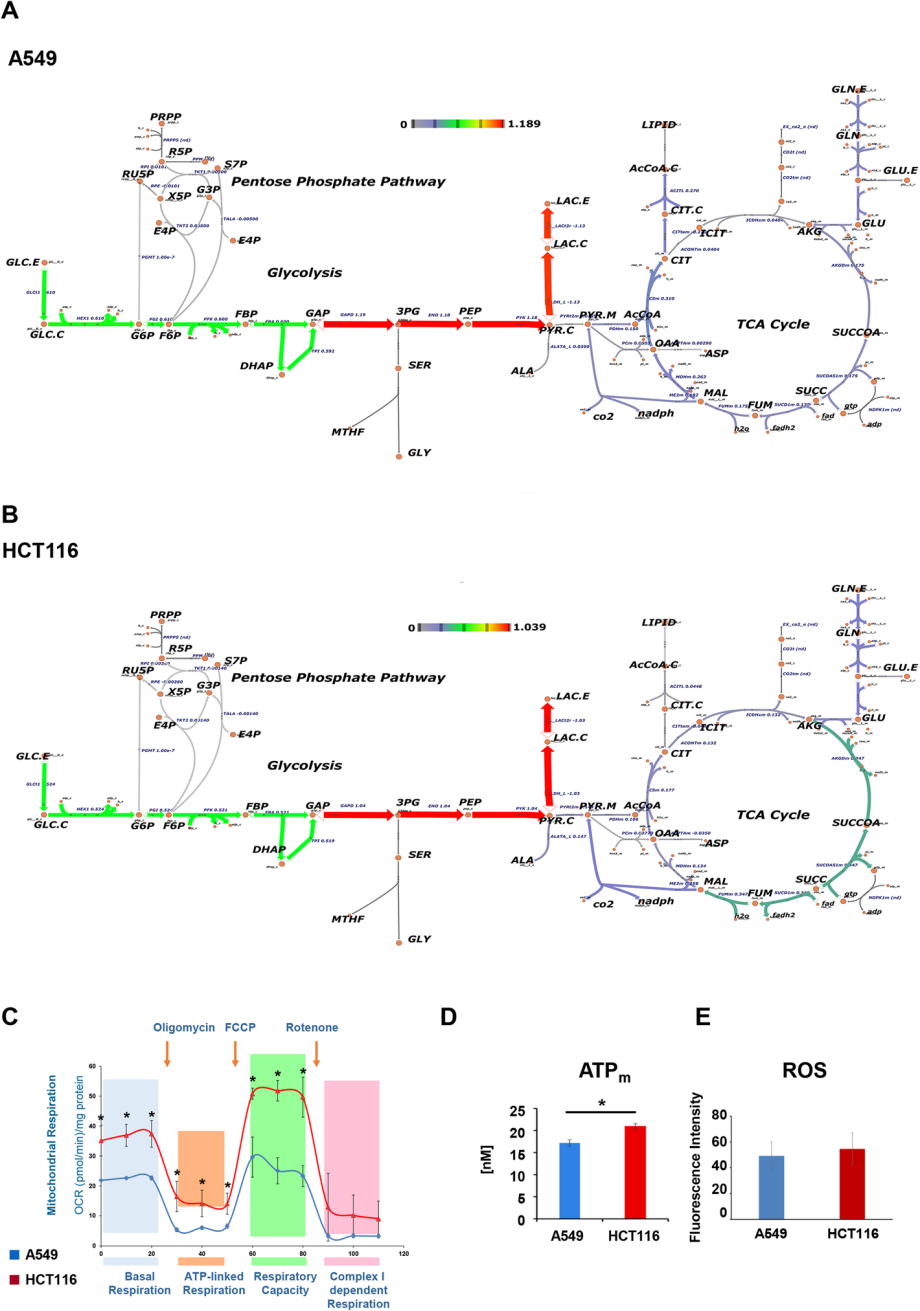
Cancer metabolic fluxes in A549 lung cancer and HCT116 colon cancer cells. **a, b** Schematic representation of central carbon metabolism with net flux values estimated by ^13^C MFA in A549 (**a**) and HCT116 (**b**) in control condition. Arrows colors (red color = up flux, violet = down flux) and thickness represent the significantly different fluxes (based on 95% confidence intervals). **c** Mitochondrial respiration reflected by OCR levels in A549 ( ) and HCT116 ( ) cancer cells under basal conditions or following the addition of oligomycin (1 μM), the uncoupled FCCP (1 μM), or the electron transport inhibitor Rotenone (0.5 μM) (*n* = 5). **d**, **e** Mitochondrial ATP (**d**) and intracellular ROS levels (**e**) were measured by Seahorse assay and DCFDA staining, respectively, in A549 ( ) and HCT116 ( ) maintained in standard growth medium for 48 h. Error bars indicate SD (*n* = 3)

Oncogenic *K-Ras* may alter glucose and glutamine metabolism to sustain enhanced cell proliferation [13, 26]. Although both A549 and HCT116 cancer cells harbor a constitutively activated *K-Ras* gene, they show a distinctive metabolic phenotype, as evidenced by a combination of physiological and metabolic analyses (Figs. 1 and 2).

Compared with HCT116 cells, A549 cells are more sensitive to the limitation of glucose (Glc) and glutamine (Gln) (Fig. 1a). In A549 cells, both glucose and glutamine limitations severely restrict the proliferation after 24 h. In HCT116 cells, glucose limitation reduces growth after 48 h, while glutamine limitation has little or no effect on cell growth during the first 72 h. The rates of glucose consumption and lactate production are similar in the two cell lines (Fig. 1b). HCT116 cells secrete less lactate than A549 cells, but the difference is not statistically significant.

Compared with HCT116 cancer cells, the A549 cell line presents a distinct metabolic profile (Fig. 1c), with significantly higher levels of metabolites involved in amino acid metabolism (such as alanine-aspartate-glutamate, arginine-proline-cysteine, and methionine), glycolysis, pentose phosphate pathway (PPP) and TCA cycle. The HCT116 cell line, instead, shows an increase in metabolites involved in the maintenance of the redox status, such as folate, glutathione, and polyamine metabolism (Fig. 1c). Pathway enrichment analysis of metabolites whose level differ at least 2-fold in a statistically significant manner between the two cell lines provides a first indication that nutrient limitation up-regulates fatty acid oxidation, amino acid metabolism, and glycolysis/gluconeogenesis in A549 cells (in comparison with HCT1116 cells) (Fig. 1d). Compared with the A549 cell line, HCT116 cells show increased levels of metabolites involved in the maintenance of the redox status, such as folate metabolism (Fig. 1e).

Enrichment analysis is not sufficient to unambiguously identify differentially regulated pathways in a pair-wise comparison because some metabolites (such as ADP or coenzyme A) are present in many pathways. Thus, their presence within a list of differentially regulated metabolites is sufficient to enrich for that pathway. Metabolic flux analysis allows to estimate intracellular metabolic fluxes (Supplementary Tables 1–2 and Supplementary file 1). The technique uses relative incorporation of uniformly labeled glucose ([U-^13^C_6_]Glc) and glutamine ([U-^13^C_5_]Gln) tracers in the different metabolites (Figure S1A) to constrain an elementary metabolite unit (EMU)-based algorithm. The resulting computed metabolic fluxes (Fig. 2a and b) indicate that A549 and HCT116 show a similar sustained glycolytic flux from glucose to extracellular lactate (Fig. 1b). Glutamine-derived alpha-ketoglutarate (AKG) can enter the TCA cycle and follow either a canonical clockwise direction, whose first step is its conversion to succinyl CoA, or a reverse route back to citrate, whose final destiny is lipid production. A549 cells also follow the glutamine reductive carboxylation route, as previously reported in lung cancer cells [27]. Instead, HCT116 cells show a slightly higher flux through the forward TCA cycle route, suggesting a preferential use of respiration (Fig. 2a and b). This interpretation is confirmed by Seahorse analysis that indicates that HCT116 cells have higher basal and maximal (i.e., uncoupler-induced) respiration as compared to A549 cells (Fig. 2c), consistent with the increased mitochondrial ATP (Fig. 2d). ROS levels do not change significantly between the two cell lines (Fig. 2e), suggesting that differential mitochondrial activity is not due to the dysfunction of mitochondrial complexes. In summary, these results indicate that the A549 and HCT116 cancer cell lines present a detectably different metabolic rewiring, the colon-derived HCT116 cells being more respiratory than the lung-derived A549 cells.

### Combinatorial treatment with glutaminase and PI3K/aldolase inhibitors blocks the proliferation of xenografts induced by A549 lung and HCT116 colon human cancer cells

**Fig. 3:**
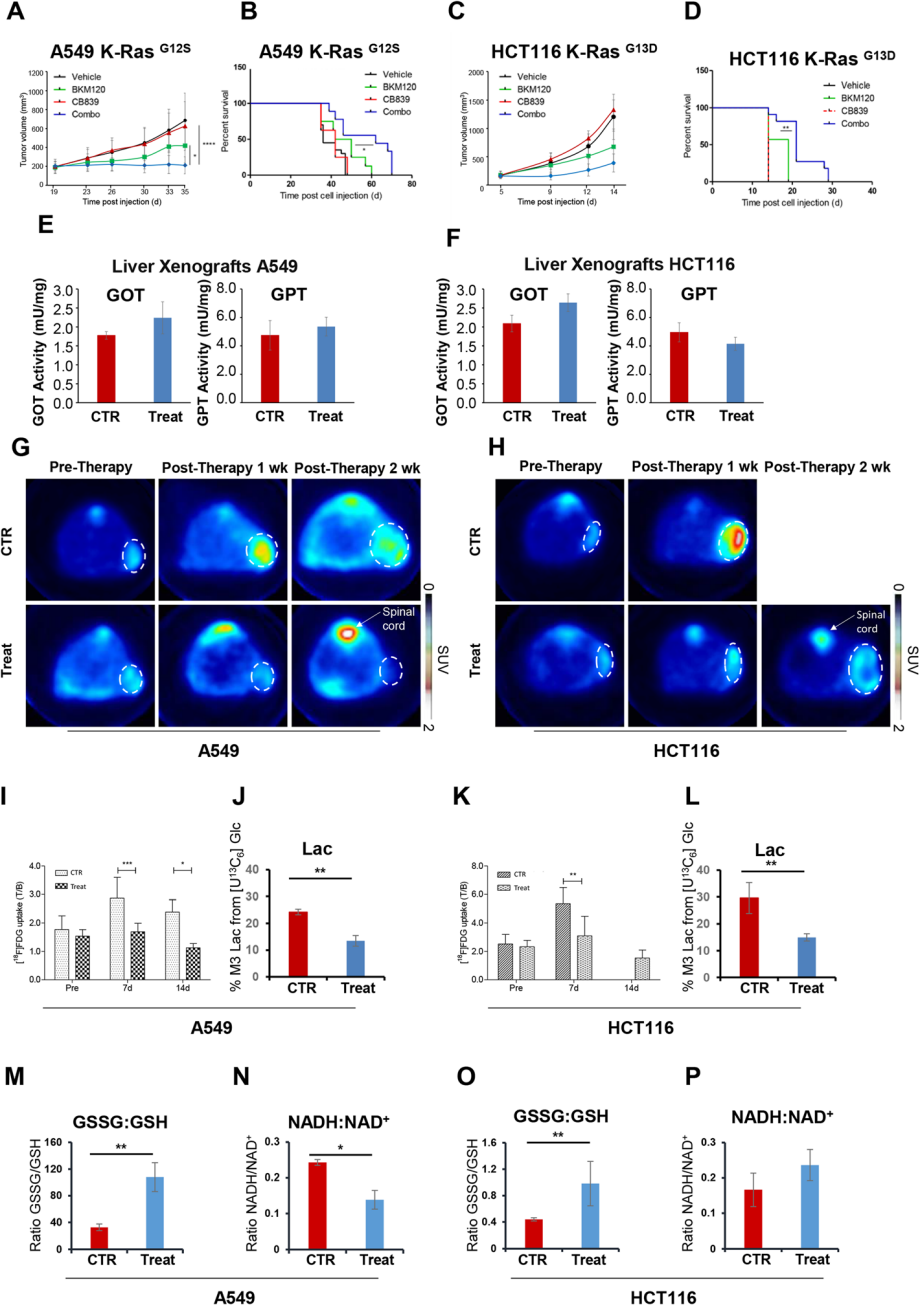
Analysis of A549 lung tumors and HCT116 colon tumors under combinatorial treatment. **a** and **c** A549 (**a**) and HCT116 (**c**) tumor volume measured by caliper in mice treated for 14 days with vehicle (CTR ), or BKM120 ( ), or CB-839 ( ), or a combination of BKM120 plus CB-839 ( ). **b** and **d** Kaplan–Meier survival curves of A549 lung (**b**) and HCT116 colon (**d**) tumor-bearing mice. The combined treatment significantly increases survival, as compared with BKM120 alone (*p* < 0.05). **e**, **f** Evaluation of hepatotoxic effects of drugs by assessing aspartate transaminase (GOT) and alanine transaminase (GPT) on the liver from A549 (**e**) and HCT116 (**f**) tumor-bearing mice exposed to the combinatorial treatment compared to CTR. **g**, **h** Representative transaxial [^18^F]FDG PET images of A549 (**g**) and HCT116 (**h**) tumors in CTR and combined-treatment mice performed before and after drug administration for 1 or 2 weeks. The color scale is expressed as Standardized Uptake Value. **i** and **k** [^18^F]-FDG uptake in A549 (**i**) and HCT116 (**k**) tumors exposed to the combined treatment (Treat) compared with CTR, expressed as tumor to background ratio (T/B). **j** and **l** Lactate labeling evaluated by [U-^13^C_6_]Glc infusion in A549 (**j**) and HCT116 (**l**) xenograft mice exposed to the combined treatment compared to CTR and analyzed by GC-MS. **m** and **o** GSSG/GSH ratio in CTR ( ) or combined treatment ( ) in A549 (M) and HCT116 (**o**) xenografts based on relative abundance obtained by LC-MS analysis. **n** and **p** NADH/NAD^+^ ratio in CTR ( ) or combined treatment ( ) in A549 (N) and HCT116 (P) xenografts based on relative abundance obtained by LC-MS analysis. *p < 0.05; **p < 0.01; ***p < 0.001; ****p < 0.0001

Cancer cells use both glucose and glutamine for their metabolic needs [1]. We tested the effect of single or combined inhibition of glucose utilization and glutamine metabolism on tumor formation in nude mice. Preliminary experiments showed that BKM120 (a pan-PI3K inhibitor that decreases glucose consumption by reducing the release of active aldolase from actin cytoskeleton) and CB-839 (a reversible non-competitive allosteric GLS inhibitor) are active on both A549 and HCT116 cancer cell lines, eliciting the expected biochemical effect, i.e., a decrease in aldolase activity and intracellular glutamate concentration (Figure S1B and C). When tumors reached a volume of 130–150 mm^3^, mice were treated with either the vehicle (CTR), or BKM120 (50 mg/kg) [19, 28], or CB-839 (200 mg/kg) [1, 12], or their combination. Although the CB-839 glutaminase inhibitor was not effective on tumor growth of both xenografts when administered as monotherapy, its combined utilization with the BKM120 aldolase inhibitor had a synergistic effect by increasing the tumor growth inhibition (TGI) from 37 to 70% (*p* < 0.05) and from 47 to 66% (*p* < 0.05) for A549 and HCT116 xenografts, respectively, as indicated by caliper measurement over a 14 days window (Fig. 3a and c) and by post-mortem weight determination (Figure S1D and E). Moreover, the continuous combinatorial administration of the two drugs significantly increases the survival of both tumor mice models (Fig. 3b and d): the median survival of HCT116 tumor-bearing mice raises from 14 to 21 days (*p* < 0.0001), whereas the median survival of A549 tumor-bearing mice shifts from 36 to 62 days (*p* < 0.01). Treated mice well tolerate the combined treatment, since they show neither weight loss (Figure S1D and E) nor alteration in aspartate transaminase (GOT) and alanine transaminase (GPT) activity, standard indicators of hepatic function (Fig. 3e and f). Combined drug treatment of A549 and HCT116 xenografts significantly reduces the uptake of [^18^F] FDG (Fig. 3g–i and k, circled area, while the unmarked area shows uptake in the spinal cord with its enclosing vessels). The combined treatment also decreases lactate production from labeled glucose (Fig. 3j and l) [29] and reverses the reduced/oxidized ratio of glutathione (Fig 3m and o). The NADH/NAD^+^ ratio is also significantly decreased by drug treatment in A549 xenografts, while showing a moderate, not significant, increase in HCT116 xenografts (Fig. 3n and p).

Association (connection) of nodes in a network can be expressed using the Spearman’s correlation index so that the elements of the resulting connectivity matrix are in the interval [−1, 1] [30]. Network connectivity analysis among tumor volume (taken as a relevant phenotypic descriptor of the xenograft aggressiveness) and all possible pairs of metabolites (data obtained from metabolic profiling of A549 and HCT116 xenografts tumor performed by mass spectrometry analysis) identifies a total of 838 statistically significant interactions in untreated xenografts (Figure S1F). A total of 528 and 266 interactions are specific for A549 and HCT116 xenografts, respectively (Figure S1F). Only 54 (about 6%) are common to both xenografts. The metabolite interactions in common between the two cell lines do not include any interaction with tumor volume (Figure S1G).

**Fig. 4:**
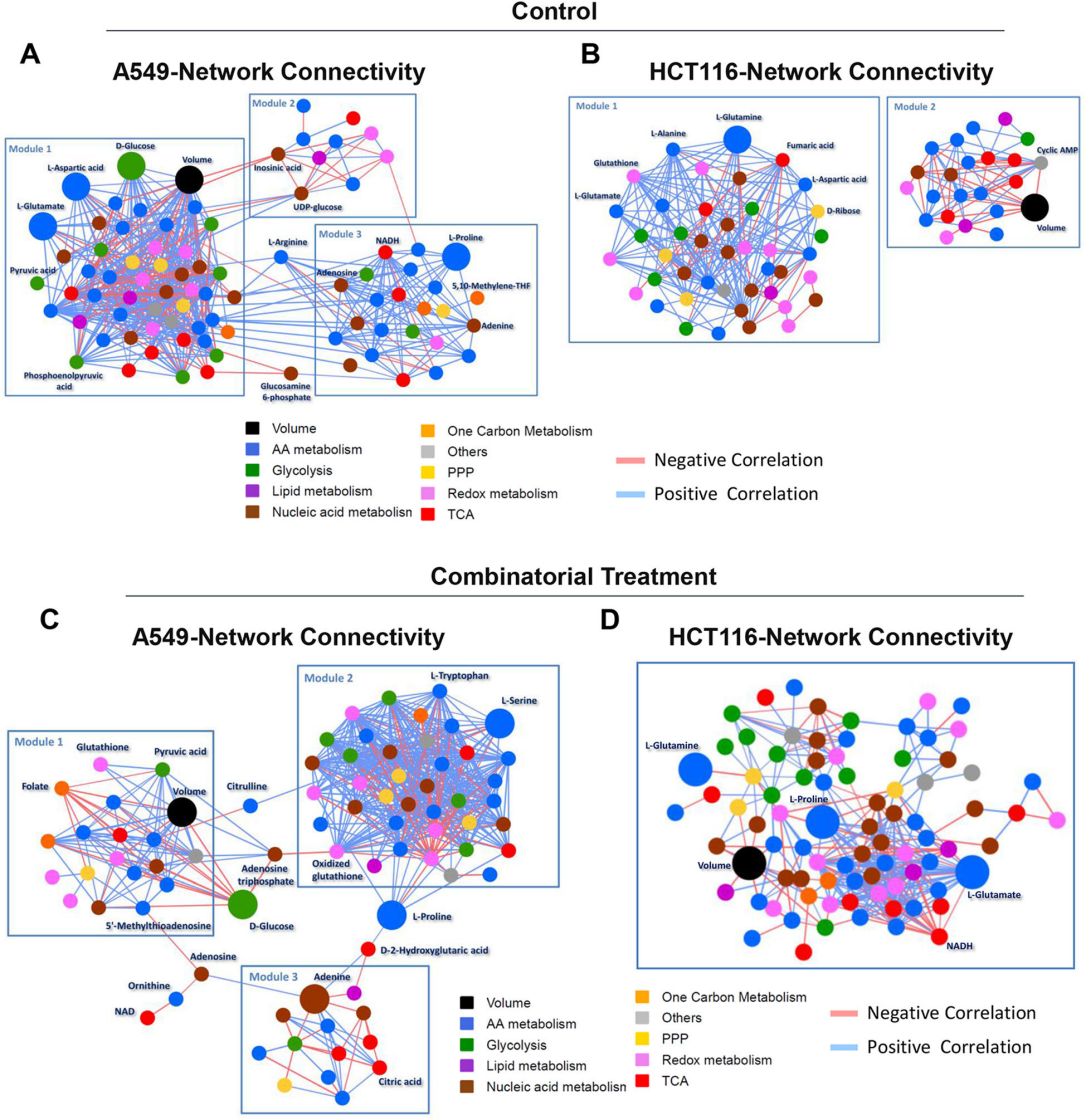
Connectivity analysis between metabolism and tumor growth of lung tumor and colon tumor-bearing mice. **a**, **b** The network of connections of A549 (**a**) and HCT116 (**b**) xenografts tumor in the control condition. **c**, **d** The network of connections of A549 (**c**) and HCT116 (**d**) xenografts tumor under combined treatment condition. All networks are specific for each tissue and obtained by keeping only the connections not shared between the two tissues. In each network, nodes represent metabolites. Nodes color corresponds to the different metabolic classes and tumor size, whereas the color of the edges indicates positive (light blue) or negative (light red) correlation. A greater size highlights nodes of interest

Using the Cytoscape plugin MCODE, we could extract locally dense modules of connected metabolites from the whole A549 and HCT116 connectomes (Fig. 4a and b). In the untreated A549 xenografts (Fig. 4a), we find three modules connected either directly or through two bridging nodes (arginine and glucosamine-6 phosphate). In module 1, glucose positively correlates with metabolites involved in amino acid metabolism and tumor volume (Fig. 4a). The combined drug treatment of A549 cells induces a strong connectivity rewiring (Fig. 4c), the most apparent change being the inversion in the polarity of the connection between glucose and tumor volume: the connection is positive in control samples and turns out negative in treated samples (Fig. 4a vs. c). In untreated HCT116 (Fig. 4b), we find two non-connected modules. In module 2, tumor volume negatively correlates with the second messenger cAMP. In module 1, glutamine is a local hub showing an average positive, strong correlation with metabolites involved in the redox and nucleotide metabolism (Fig. 4b). A single module is present in the drug-treated xenograft (Fig. 4d), indicating that the combined drug treatment induces severe metabolic rewiring in the lung xenograft. Glutamine loses its centrality, becoming a poorly connected, peripheral node.

### Rewired metabolism of treated cells results from cellular attempts to counteract the effect of inhibitory drug treatment

**Fig. 5:**
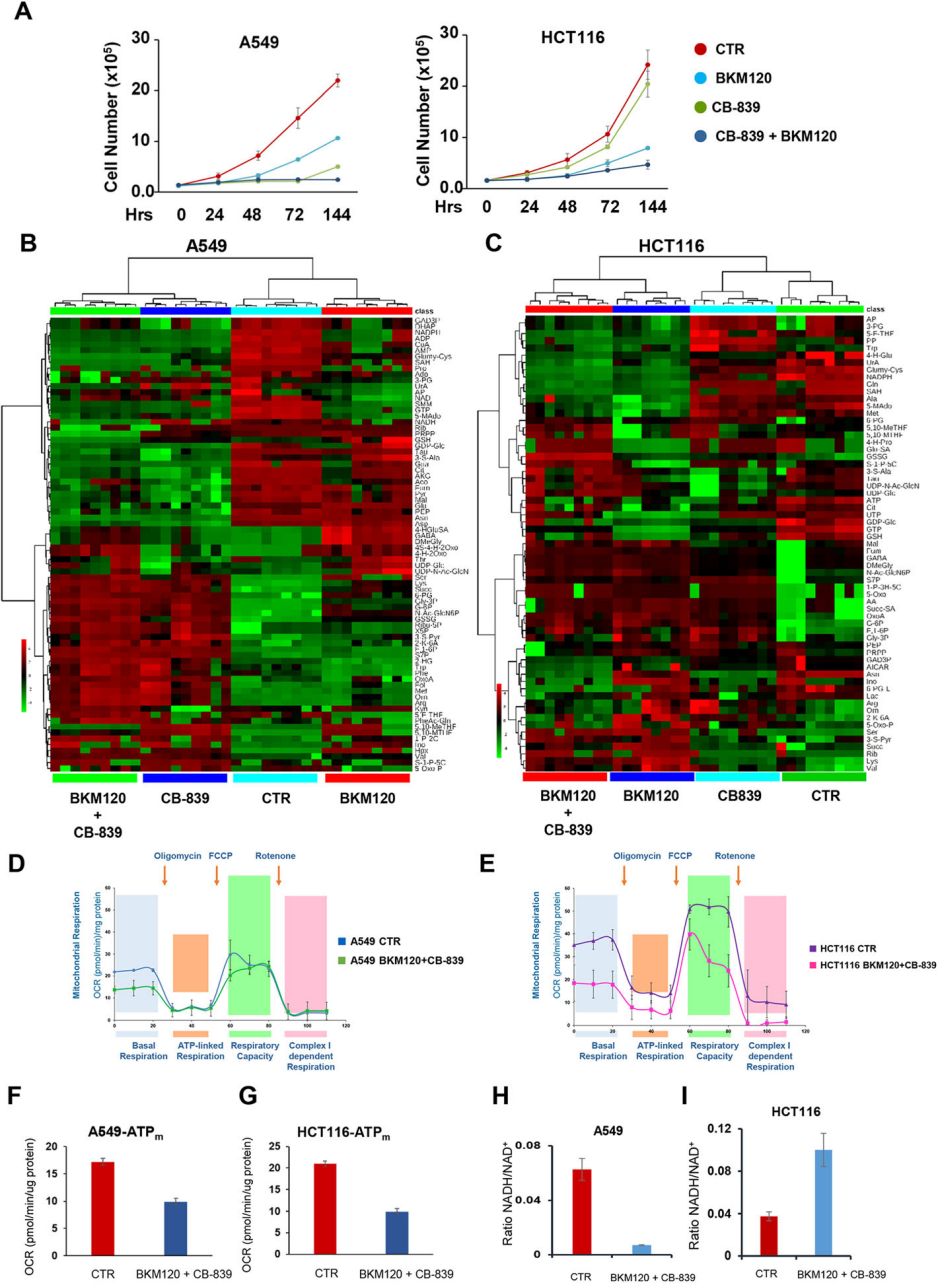
Combined glycolysis and glutamine metabolism inhibitory drugs induce growth arrest in human cancer cell lines. **a** Proliferation curves of A549 (left panel) and HCT116 (right panel) incubated with BKM120 ( ), CB-839 ( ), or BKM120 + CB-839 ( ) versus CTR ( ), collected and counted at the indicated time points. **b**, **c** Untargeted metabolic analysis in A549 (**b**) and HCT116 (**c**) cancer cells. Hierarchical clustering heatmaps show significantly different intracellular metabolites in the four experimental conditions assessed by LC-MS and GC-MS. (*p* ≤ 0.05). **d, e** Mitochondrial respiration levels in A549 CTR ( ) or under combinatorial treatments ( ) (**d**), and in HCT116 CTR ( ) or under combinatorial treatments ( ) (**e**), under basal conditions or following the addition of oligomycin (1 μM), the uncoupler FCCP (1 μM) or the electron transport inhibitor Rotenone (0.5 μM) (n = 5). **f**, **g** Mitochondrial ATP level reflected by OCR levels in A549 (**f**) and HCT116 (**g**) CTR ( ) or under combinatorial treatments ( ), under basal conditions or following the addition of oligomycin (1 μM), the uncoupler FCCP (1 μM) or the electron transport inhibitor Rotenone (0.5 μM) (n = 5). **h**, **i** NADH/NAD^+^ ratio in CTR ( ) or under combined treatment ( ) in A549 (H) and HCT116 (I) based on relative abundance obtained by LC-MS analysis

Even though some differences in the behavior of cell lines and cognate xenotransplants are expected [13], cell lines allow easier access to labeling techniques that, in turn, enable to follow metabolic rewiring in more detail. In 2D-cultures, the combined treatment with BKM120 plus CB-839 is also more effective than the single treatments, although the combinatorial drug administration is less dramatic than observed in xenografts, at least in the case of the HCT116 cell line. In both cell lines, metabolic profiles of cells treated with both BKM120 and CB-839 cluster with the metabolite profile of cells treated with the drug that mostly affects proliferation (Fig. 5a–c).

Metabolic profiling identifies significantly higher levels of metabolites involved in the first step of glycolysis, pentose phosphate pathway (PPP), and amino sugar metabolism (Figs. 5b and S2A–B), suggesting an attempt to activate alternative glucose-dependent pathways and ROS scavengers metabolic pathways such as one carbon and methionine cycle (Figs. 5c and S2C–D) in ROS-stressed cells under combined treatment (Figure S2E and F).

We then used labeling with [U-^13^C_6_]Glc and [U-^13^C_6_]Gln in A549 and HCT116 cells treated with both drugs (Figures S3A and C, blue and purple dots; S3B and D, green and violet dots, respectively). MFA indicates that the combinatorial drug treatment of A549 cells induces a significant flux reduction in the upper part of glycolysis, accompanied by an increased F6P flux towards the PPP with a significant re-feeding of ribulose 5 phosphate (Ru5P) to G6P (Figures S3C and S4A). As a result, the flux in the lower part of glycolysis is also severely reduced, leading to a reduced flux towards lactate production and secretion. The increased fluxes may partly support the residual lactate flux through the PDH and malic enzymes (Figure S4A). The citrate produced from the AcetylCoA flux is diverted to the ACL enzyme, even more heavily than in untreated cells, while Oaa is primarily used in the formation of Asp. Combined with the decreased Gln oxidation resulting from GLS inhibition, these metabolic changes lead to a decrease of basal mitochondrial respiration (Fig. 5d) and mitochondrial ATP production (Fig. 5f).

The combined treatment impacts less profoundly on the glycolytic flux of HCT116. The flux to lactate remains sustained, and the PPP pathways flux is much less activated than in A549 cells (Figures S3B and S4B). Similar to what is observed in A549 cells, the combinatorial drug treatment induces a significant reduction of glutamine oxidation flux via the TCA cycle as compared with CTR (Figure S4B), leading to a decrease of basal mitochondrial respiration (Fig. 5e) and mitochondrial ATP production (Fig. 5g). Taken together, the less pronounced effect of the combined treatment on the second step of glycolysis observed in HCT116 cells, and the higher flux of malic enzyme are all metabolic mechanisms able to increase NADH levels (Fig. 5h–i), which in turn may also affect epigenetic regulation leading to survival pathways [31].

**Fig. 6:**
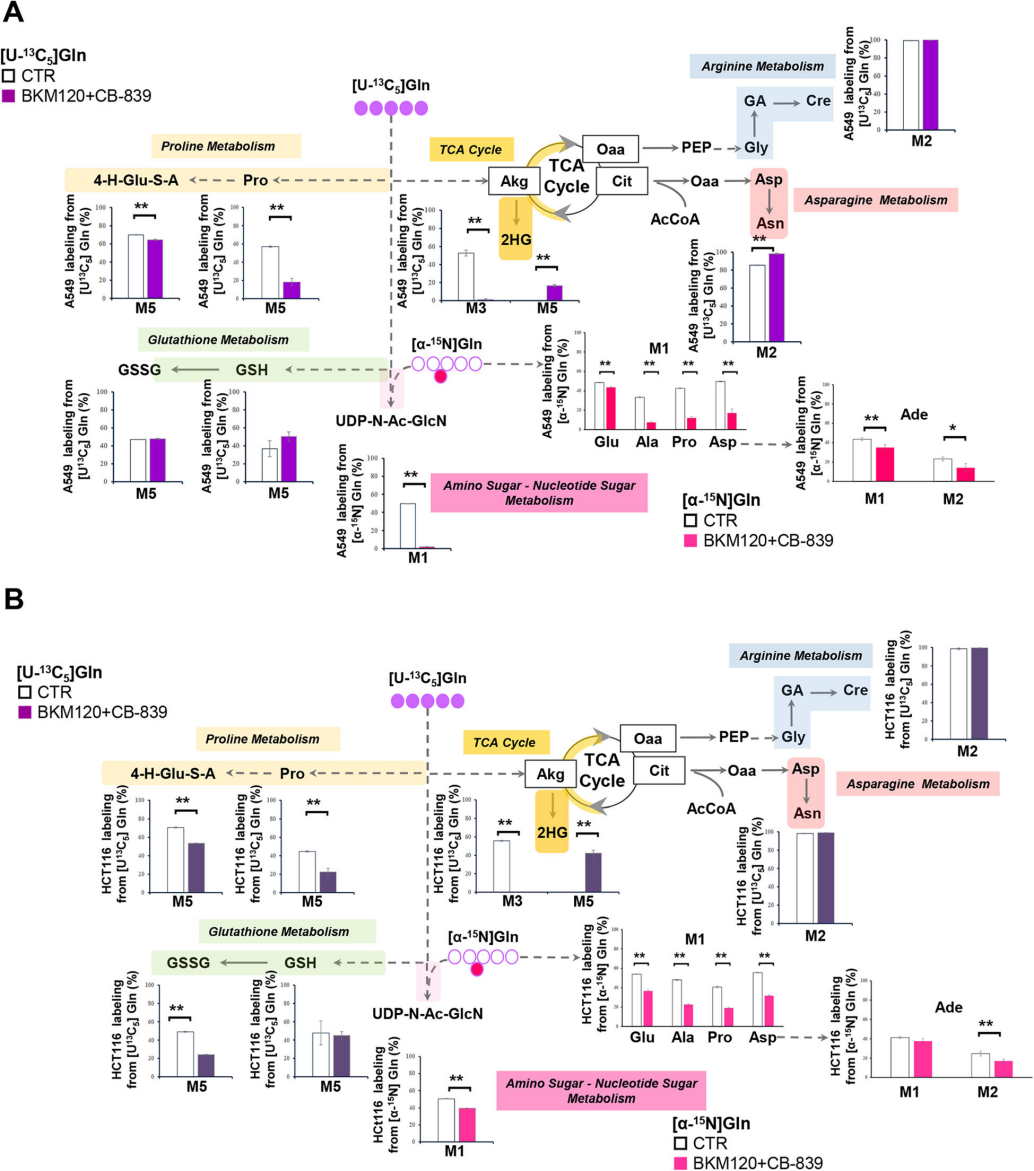
Analysis of alternative pathways in lung cancer and colon cancer cells under combinatorial drug treatment. **a, b** Schematic representation and percentage isotope labeling enrichment of metabolites from [U-^13^C_5_]Gln and [α^15^N]Gln in A549 (**a**) and HCT116 (**b**) cancer cells

To better investigate the metabolic plasticity of A549 and HCT116 cancer cell lines and to assess their tendency to escape combinatorial drug treatment through drug-dependent metabolic reprogramming, we performed a more in-depth isotope labeling analysis. The labeling of metabolites involved in amino sugar metabolism (M4 UDP-Glc, M6-N-Ac-GlcN-1P, and M6-UDP-N-Ac-GlcN) and PPP (M5-Ru5P) coming from [U-^13^C_6_]-Glc, observed in both A549 (Figure S3C) and HCT116 cells (Figure S3D) under combined drug treatment, confirms an attempt to reprogram glucose metabolism to fulfill anabolic demands [32]. Ribulose 5 phosphate M5-Ru5P labeling indicates an independent activation of PPP in all experimental conditions, while the significantly decreased labeling of hypoxanthine (Hpx, Figure S3C) correlates with reduced proliferation since it is not observed in HCT116 cells treated with CB-839 (Figs. 5a and S3D). Moreover, the significantly lower adenine labeling, coming from [α-^15^N]-Gln (Fig. 6a and b, fuchsia dots), found in cancer cells under combined treatment as compared with CTR and single treatments, is consistent with the strong effect of combined treatments on cell proliferation arrest (Fig. 5a), thus confirming the in vitr*o* efficacy of the inhibitors [9].

Labeling with [U-^13^C_5_]Gln and [^15^N]Gln stable isotopes (highlights the activation of glutamine alternative metabolic pathways in both cancer cell lines Fig. 6a and b, violet, and pink dots, respectively). Both Asn and creatinine (Cre) derive from the forward utilization of glutamine in the TCA cycle and by the coordinated activity of ATP citrate lyase (ACLY), pyruvate carboxykinase (PCK), and glycine amidinotransferase (AGAT) N-guanidinoacetate methyltransferase (GAMT) (Fig. 6a and b). The combination treatment does not significantly affect the labeling of M2-Cre (Fig. 6, light blue color), but increases levels of labeled Asn (Fig. 6a, pink color), an amino acid promoting cancer cell proliferation [33] and survival [3]. The combined treatment also increases Gln-derived labeling of glutathione (M2-GSH and GSSG) (Fig. 6a and b, green color) and metabolites involved in proline metabolism (yellow color), such as M5-Pro and M5 4-hydroxy glutamate semialdehyde (4-H-Glu-S-A), in both cell lines.

The amino-nucleotide sugar metabolism (pink color) also uses glutamine as an amino group donor to glucose-6-phosphate, to produce glucosamine-6-phosphate. Hexosamine biosynthesis requires a coordinated utilization of glucose and glutamine, leading to glycosylation of signal transduction components regulating cell growth and proliferation [34]. A549 cells lose this coordination under combinatorial and single CB-839 treatment, as shown by the M1 UDP-N-Ac-GlcN labeling derived from [^15^N]Gln (Fig. 6A, fuchsia dots). We also found significantly lower labeling for some metabolites (such as Glu, Ala, Pro, and Asp) in both human cancer cell lines exposed to combined treatments compared with CTR (Fig. 6a and b).

Consistent with these metabolic observations, RNA-seq analysis shows a different response of both cell lines to the combined treatment also at the transcriptional level. HCT116 cells show only 31 genes with a statistically significant expression change in response to the combined treatment (Supplementary File 2). Gene enrichment analysis highlights that down-regulated genes (*n* = 28 genes) mainly participate in metabolic processes, such as organic acid, carboxylic acid, fatty acid, pyruvate, propionate, acetate, and acetyl-CoA metabolisms (Figure S4E). The three up-regulated genes are involved in cell cycle and adhesion functions. Differently, A549 cells show a more considerable transcriptional change in response to treatments, with 73 statistically significant differentially expressed genes (29 up- and 44 down-regulated genes, Supplementary File 3). Interestingly, down-regulated genes do not enrich any particular function, except for the up-regulated aldehyde dehydrogenase 3A1 (ALDH3A1) gene. ALDH3A1 is involved in many cellular oxidative processes and is highly expressed in some human tumors [35]. Over-expression of ALDH3A1 in cancer cells correlates with an increased chemo-resistance to drugs [36, 37]. Therefore, the increased ALDH3A1 expression level in A549 lung cancer cells under combined treatment could counteract oxidative stress (Figure S4F) and provide resistance against combinatorial treatment.

## Discussion

Yadav et al. [38] recently pointed out that a “non-linear” relation between gene mutations and their phenotypic penetrance is the rule, rather than the exception, in multi-factorial diseases such as cancer. This non-linear relation results from the fact that biological functions, and their alterations in diseases, originate from the dynamic interactions of genes, proteins, and metabolites. Since metabolism integrates information from genetic, epigenetic, and environmental signals [39], metabolic alterations faithfully reflect perturbations that can modify cell physiology [40]. Using an integrated systems metabolomics approach [11] combining complementary techniques, we here show that A549 and HCT116 cells, both carrying an activated *K-Ras* oncogene, present distinct phenotypic properties.

HCT116 colon cancer cells rely on respiration more than A549 lung cancer cells (Figs. 1 and 2), in line with previous reports on different metabolic properties of cells harboring mutations in different codons of the *ras* gene [41]. The combined treatment with the glutaminase inhibitor CB-839 and the PI3K/aldolase inhibitor BKM120 consistently reduces cell growth of both A549 and HCT116 tumor xenografts, leading to a significant tumor reduction and prolonged survival without associated toxicity (Figs. 3 and S1). The combined drug treatment induces a severe metabolic rewiring, with evidence of a redox crisis. Both gamma-glutamylcysteine (Glu-Cys, a glutathione precursor) and cysteine sulfuric acid (an irreversible oxidation product of cysteine) increase in treated xenotransplants and cells cultivated in vitro, favoring the oxidized form of glutathione compared to the reduced one (Figs. 3m and o, and S4C and D). The combined drug treatment also significantly increases the ROS levels (Figure S2E and F). Labeling experiments indicate that the observed metabolic profiles depend on the primary effect of the drugs and the attempts to counteract the drug activity. By way of example, the less pronounced effect of the combined treatment on the second step of glycolysis observed in HCT116 cells, and the higher flux of malic enzyme are all metabolic mechanisms able to increase NADH levels (Fig. 3p), which in turn can also affect epigenetic regulation leading to survival pathways [31]. Moreover, the significant increase of Asp labeling, observed by using both glucose and glutamine ^13^C stable isotope tracers in A549 and HCT116 cells (Figure S3), may act as a preserving mechanism, confirming that aspartate acquisition is an endogenous metabolic limitation for the growth of some tumors [42]. Conversion of the de novo synthesized Asp to Asn would sustain ASCT2 (Slc1a5) antiport with Gln, which could feed the TCA cycle to produce ATP [43].

In a clinical setting, where a complete systems metabolomics approach may not be routinely feasible, can quantitative analyses of metabolite profiles act as a substitute for the integrated systems metabolomics analysis used in this paper?

The combined treatment dramatically disrupts overall pair-wise connectivity [44] among metabolites in A549 cells, while it has a little effect in HCT116 cells. In both cell lines, the combined treatment induces a dramatic loss of connectivity between metabolites involved in nucleic acid metabolism and metabolites involved in other pathways (Figure S5A and B, black boxes). A single administration of BKM120 or CB-839 elicits the same effect on pair-wise metabolite connectivity only if it strongly inhibits cell proliferation. These findings indicate that the connectivity of metabolites involved in nucleic acid metabolism with other metabolites is a signature of effective treatments, regardless of whether it is a single or double treatment.

The combined drug treatment results in significant up- and down-regulation of 16 and 15 metabolites, respectively, (plus NAD^+^ and NADH) involved in the redox metabolism according to the KEGG database. Among these metabolites, 14 (plus NAD^+^ and NADH) are common to both cell lines, 10 (plus NAD^+^) showing a congruent change in concentration in both A549 and HCT116 cells (Supplementary Table 3). Globally, these alterations suggest that the combined drug treatment induces a more oxidized state in both cell lines, as highlighted by the decrease in the ratio between reduced and oxidized glutathione (Figs. 3m and o, and S4C and D).

Pathway connectivity analysis indicates that in the A549 xenografts, the double treatment disrupts the connectivity of redox metabolites (bottom lines in the panels a–d, outlined in pink) with metabolites involved in other pathways (Figure S5C). In this figure, each square represents pair-wise connectivity between metabolites belonging to the color-coded pathway represented in the X and Y axes. Each square’s color indicates whether the correlation is positive (red) or negative (blue), each shade being more intense for stronger correlations. The combined treatment reverses most correlations among redox metabolites with metabolites from other pathways (color reversal) and/or makes them less significant (decreased shade intensity). A similar, although possibly less striking, effect on pathways connectivity is observed in the HCT116 xenografts (Figure S5C).

## Conclusions

Concurrent drug perturbation of glucose and glutamine utilization pathways severely reduces the growth of A549 and HCT116 xenografts and cell lines. This result is consistent with the major role that glucose and glutamine play in supporting cellular proliferation. However, results reported in Figure S6 indicate that the magnitude of the inhibitory effect can vary considerably in different cell lines and is not simply the result of *ras* mutational activation, since we observe the strongest effect in A375 melanoma cells, which have two wild type *ras* genes. In-depth metabolic analysis is therefore required to rationalize the results of the drug treatments and to develop rational guidelines for effective combinatorial drug protocols.

Our systems metabolomics analysis shows that the metabolic unbalance originated by the combinatorial drug treatment affecting both glucose utilization and glutamine metabolism may be so significant that the tumor is unable to maintain the redox homeostasis, consistently with the proposed crucial role of redox control for cancer cell growth [26, 45, 46]. These integrated analyses may be too costly, time-consuming, and even unfeasible in a clinical setting. Since connectivity analysis is performed on metabolic profiles that are faster, easier, and cheaper to obtain, it could offer a viable alternative. The tool requires testing on an appropriate panel of cancer cell lines and pre-clinical models, such as patient-derived xenografts [47] and organoids [48]. This large effort will provide the appropriate dataset required to develop an instrument able to assist in the design of combinatorial drug treatments, as well as in following their effects [44], thus opening new avenues for personalized medicine and precision oncology approaches.

## Supplementary information

**Additional file 1.** Supplementary Materials.

**Additional file 2.**

**Additional file 3.**

**Additional file 4.**

## Data Availability

All the data obtained and/or analyzed during the current study were available from the corresponding authors on reasonable request.

## Abbreviations

1,3 BPGA: 1,3-Bisphosphoglycerate
10-CHO-THF: 10-Formyltetrahydrofolate
1-P3H5C: L-1-Pyrroline-3-hydroxy-5-carboxylate
2HG: 2Hydroxyglutarate
3PGA: 3-phosphoglycerate
3-S-Ala: 3-Sulfino-L-alanine
3-S-Pyr: 3-Sulfino-pyruvate
4HGlu: 4Hydroxyglutamate
4-HGluSA: L-4-Hydroxyglutamate semialdehyde
4-Hpro: 4-Hydroxyproline
5,10-MeTHFD: 5,10-Methenyltetrahydrofolic acid
5,10-MTHFD: 5,10-Methylene-THF
5-FTHF: 5-Formimino THF
5-MTA: 5'-Methylthioadenosine
5-MTHF: 5-Methyltetrahydrofolate
5-Oxo: Pyroglutamic acid
6PGL: 6 phosphoglycerate
AcCoA: AcetylCoA
Ade: Adenine
Ado: Adenosine
Akg: α-ketoglutarate
Ala: Alanine
AMPK: Adenosine monophosphate kinase
Arg: Arginine
ArgSucc: ArgininoSuccinate
Asn: Asparagine
Asp: Aspartate
ATP: Adenosine 5'-triphosphate
Cit: Citrate
CP: Carbamoyl phosphate
Cr: Creatinine
CT: Citrulline
Cys: Cysteine
F6P: Fructose-6-phosphate
FDG: Fluorodeoxyglucose
FLT: Fluorothymidine
Fol: Folate
Fru: Fructose
G6P: Glucose-6-phosphate
GA3P: Glyceraldehyde 3-phosphate
GC/MS: Gas chromatography-mass spectrometry
Glc: Glucose
Gln: Glutamine
GLS: Glutaminase
Glu: Glutamate
Glu-Cys: Gamma-Glutamylcysteine
Gly: Glycine
GMP: Guanosine monophosphate
GSH: Glutathione
GSSG: Oxidized glutathione
Gua: Guanosine
Hcy: Homocysteine
HK: Hexokinase
Hpx: Hypoxanthine
Ile: Isoleucine
Lac: Lactate
LC3: Microtubule-associated protein 1A/1B-light chain 3
LDH: Lactate dehydrogenase
Leu: Leucine
Lys: Lysine
Met: Methionine
MSTFA: (*N*-Methyl-*N*-(trimethylsilyl) trifluoroacetamid
NAD+: NAD+
NADH: NADH
*N*-PAG: *N*-phenylacetyl-glutamine
Oaa: Oxaloacetate
Orn: Ornithine
OXPHOS: Oxidative phosphorylation
PC: Pyruvate carboxylase
PCK2: Phosphoenolpyruvate carboxykinase 2
PET: Positron emission tomography
Phe: Phenylalanine
P-pyr: Phosphopyruvate
Pro: Proline
Putr: Putresceine
Pyr: Pyruvate
Q-TOF: Quadrupole-Time of flight
R5P: Ribose 5 phosphate
Rib: Ribose
ROS: Reactive oxygen species
Ser: Serine
SMM: S-Methylmethionine
Spd: Spermidine
Succ: Succinate
Tau: Taurine
TCA: Tricarboxylic acid cycle
Thr: Threonine
TK1: Thymidine Kinase 1
Trp: Triptophane
Tyr: Tyrosine
UAC: Uric acid
Val: Valine
X5P: Xylulose 5-phosphate
Xan: Xanthine
