# Supplementary figures and images for: Disruption of redox homeostasis for combinatorial drug efficacy in K-Ras tumors as revealed by metabolic connectivity profiling
Source: Cancer Metab. 2020 Sep 29;8:22. doi: 10.1186/s40170-020-00227-4 (PMC7523077; doi:10.1186/s40170-020-00227-4)

**A**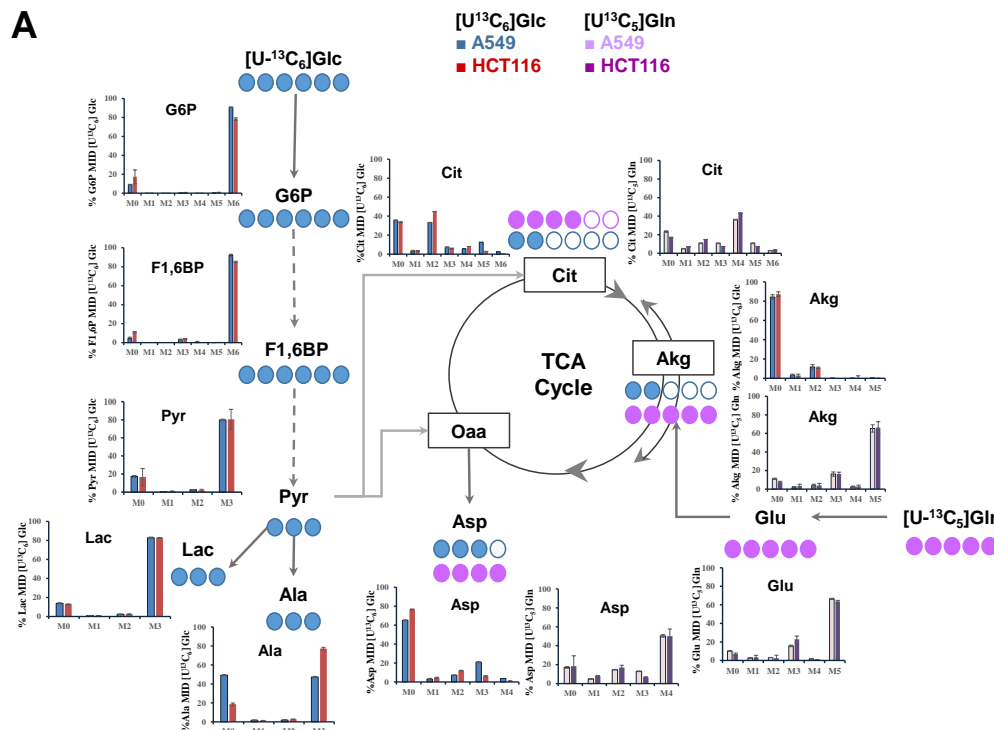**B**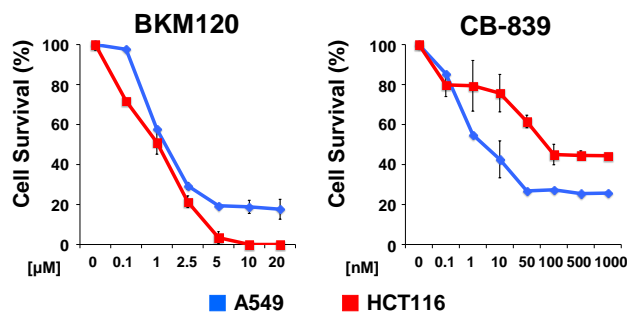**C**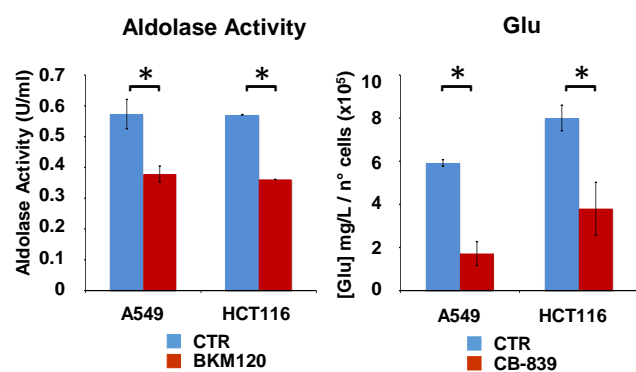**D**

A549-Tumor weight at sacrifice

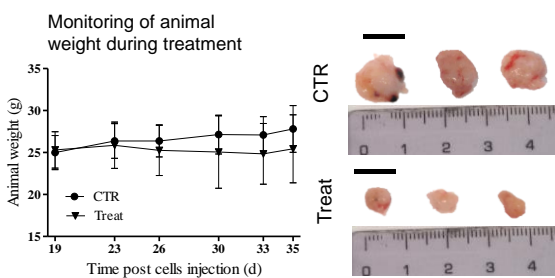**E**

HCT116-Tumor weight at sacrifice

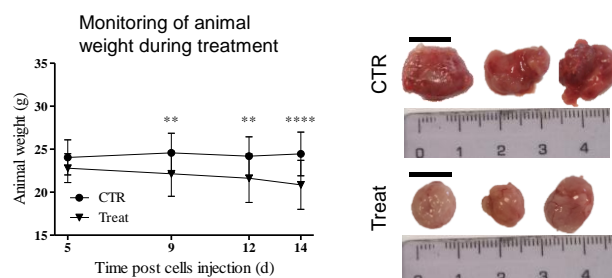**F**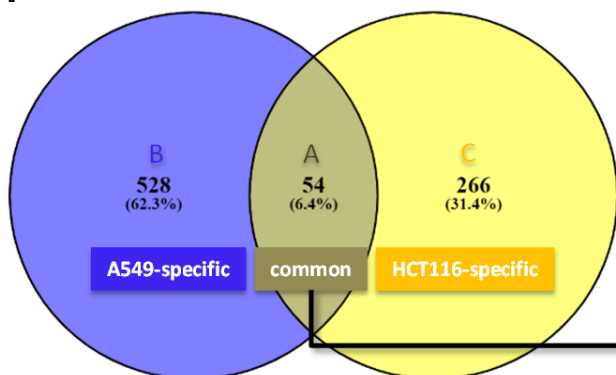**G**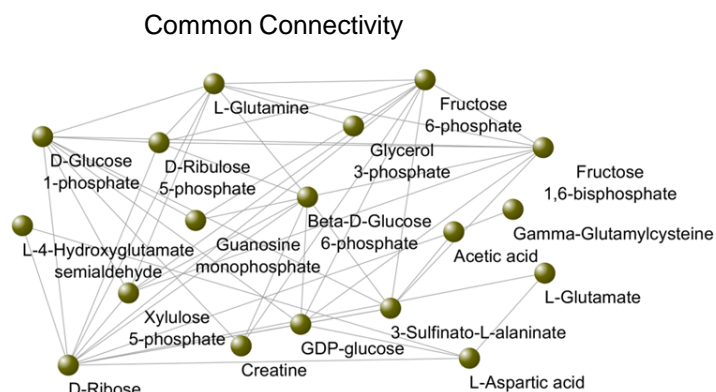**Figure S1**

Supplement: Supplementary file 1 — Additional file 1. Supplementary Materials. [file 40170_2020_227_MOESM1_ESM.zip › Figure S1.pdf]

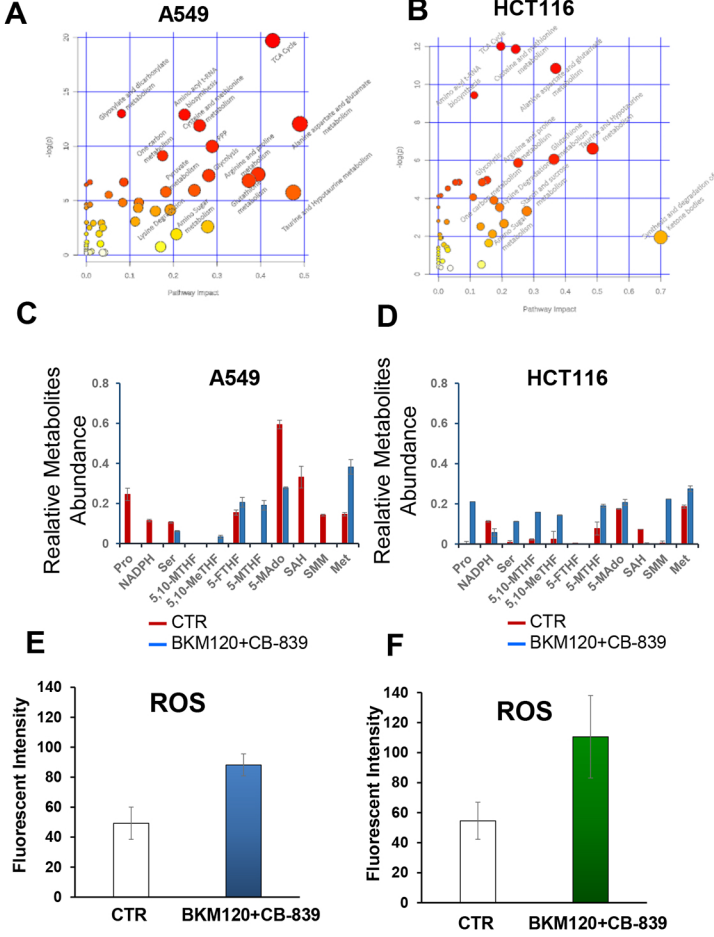

Figure S2

Supplement: Supplementary file 1 — Additional file 1. Supplementary Materials. [file 40170_2020_227_MOESM1_ESM.zip › Figure S2.pdf]

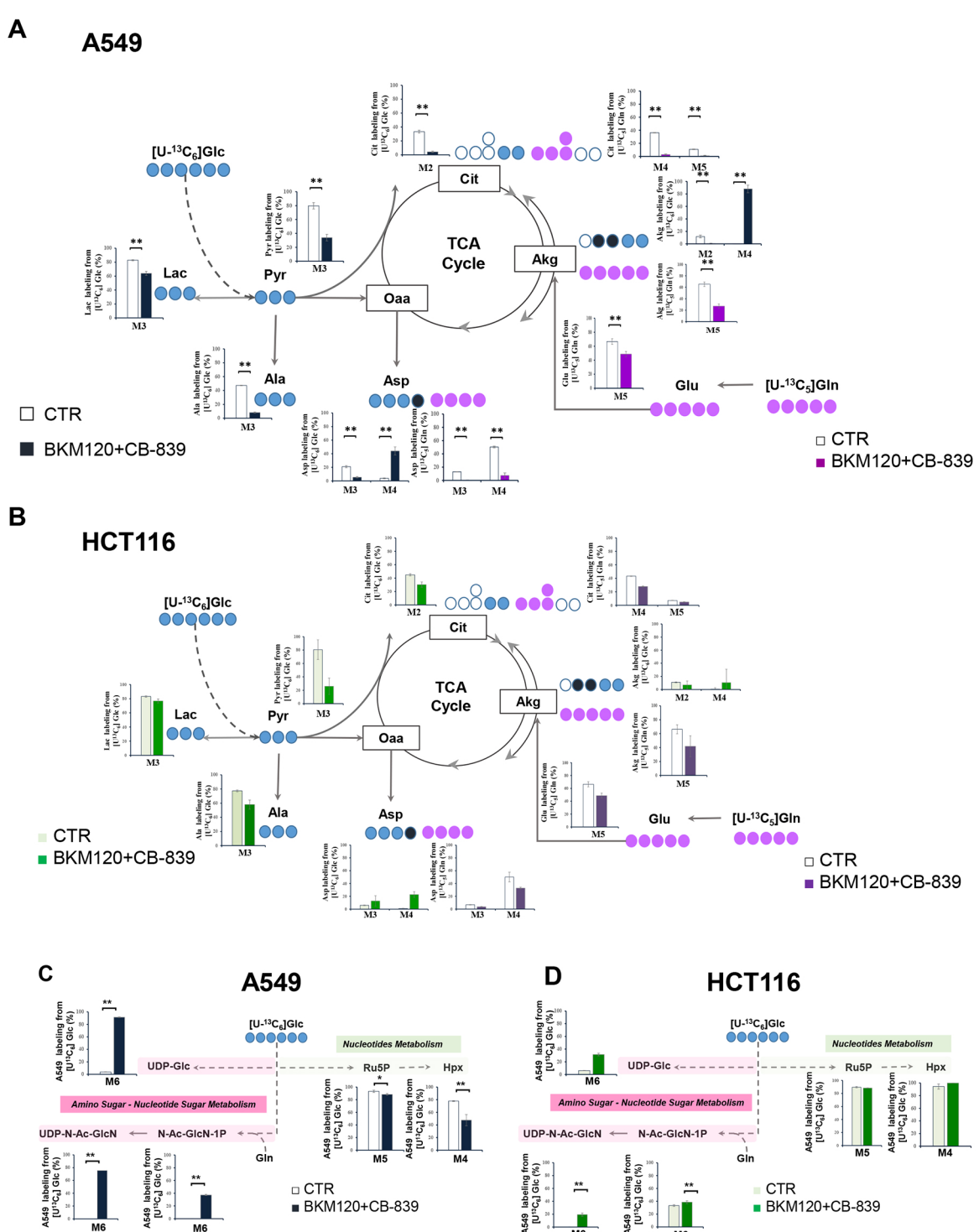

Figure S3

Supplement: Supplementary file 1 — Additional file 1. Supplementary Materials. [file 40170_2020_227_MOESM1_ESM.zip › Figure S3.pdf]

**A A549 BKM120+CB-839**

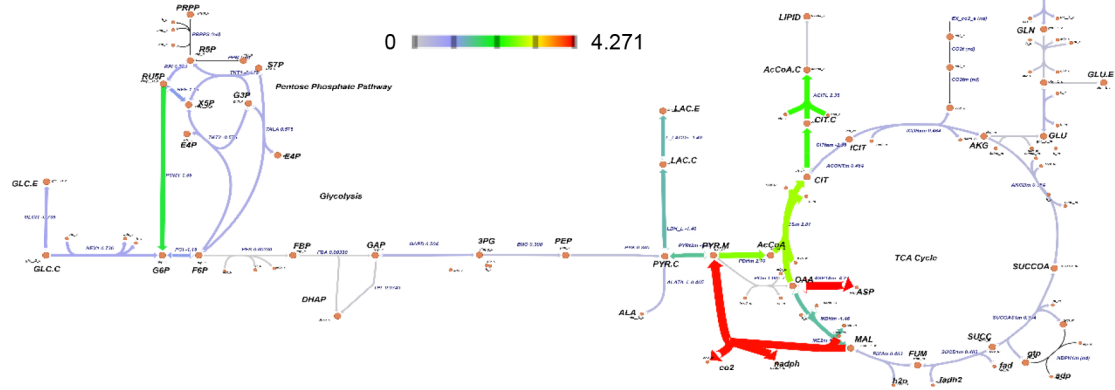

**B HCT116 BKM120+CB-839**

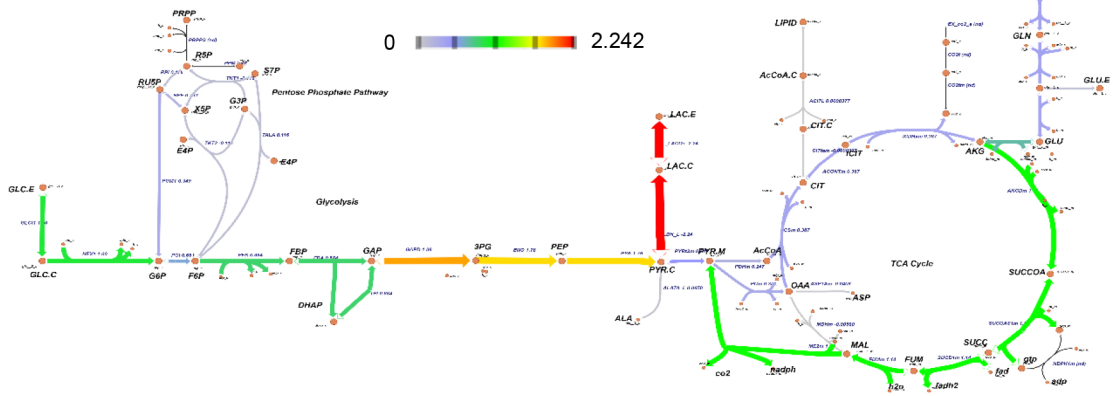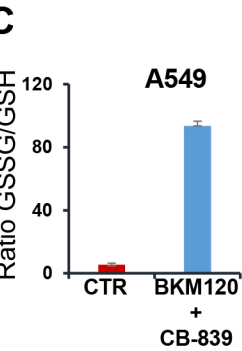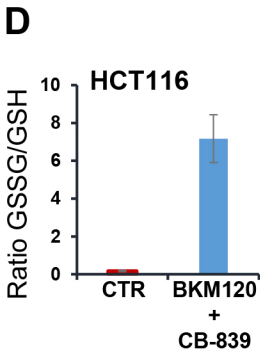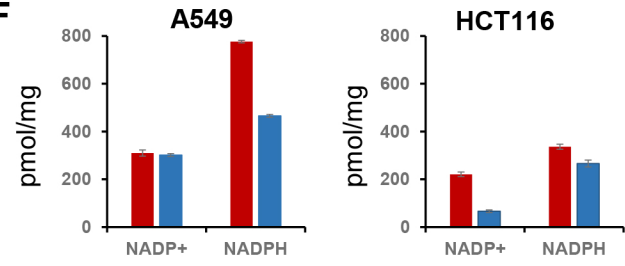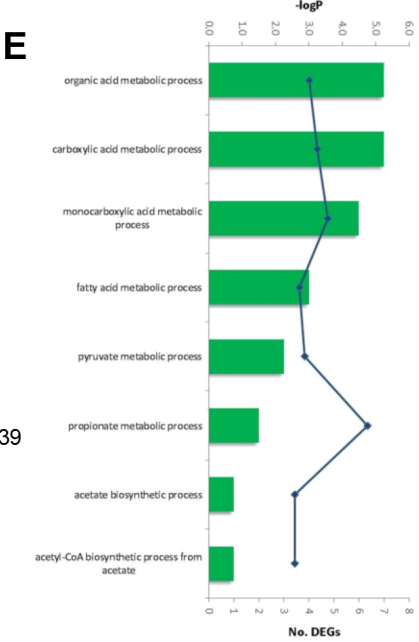

**Figure S4**

Supplement: Supplementary file 1 — Additional file 1. Supplementary Materials. [file 40170_2020_227_MOESM1_ESM.zip › Figure S4.pdf]

**A****A549**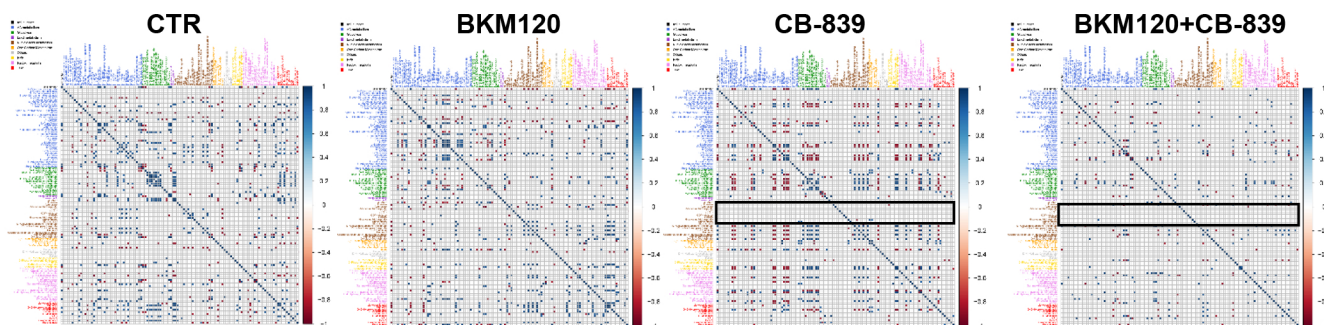**HCT116****B**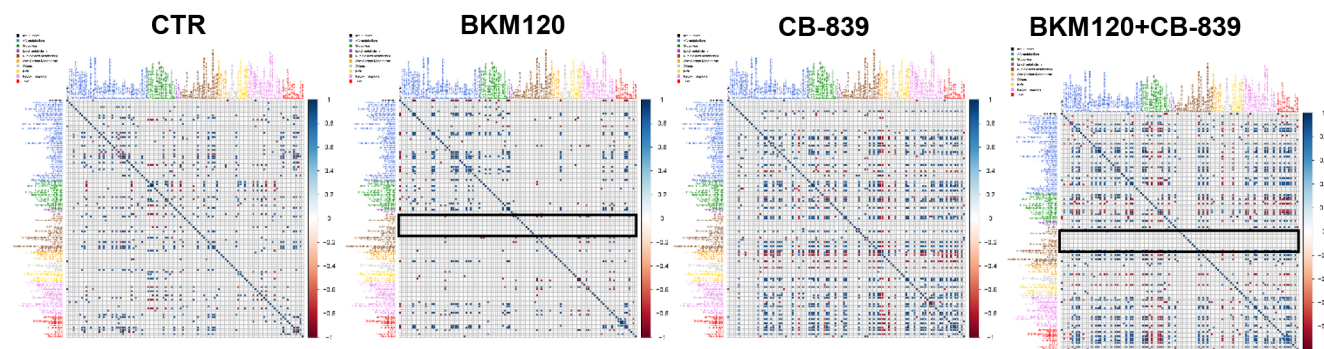**C**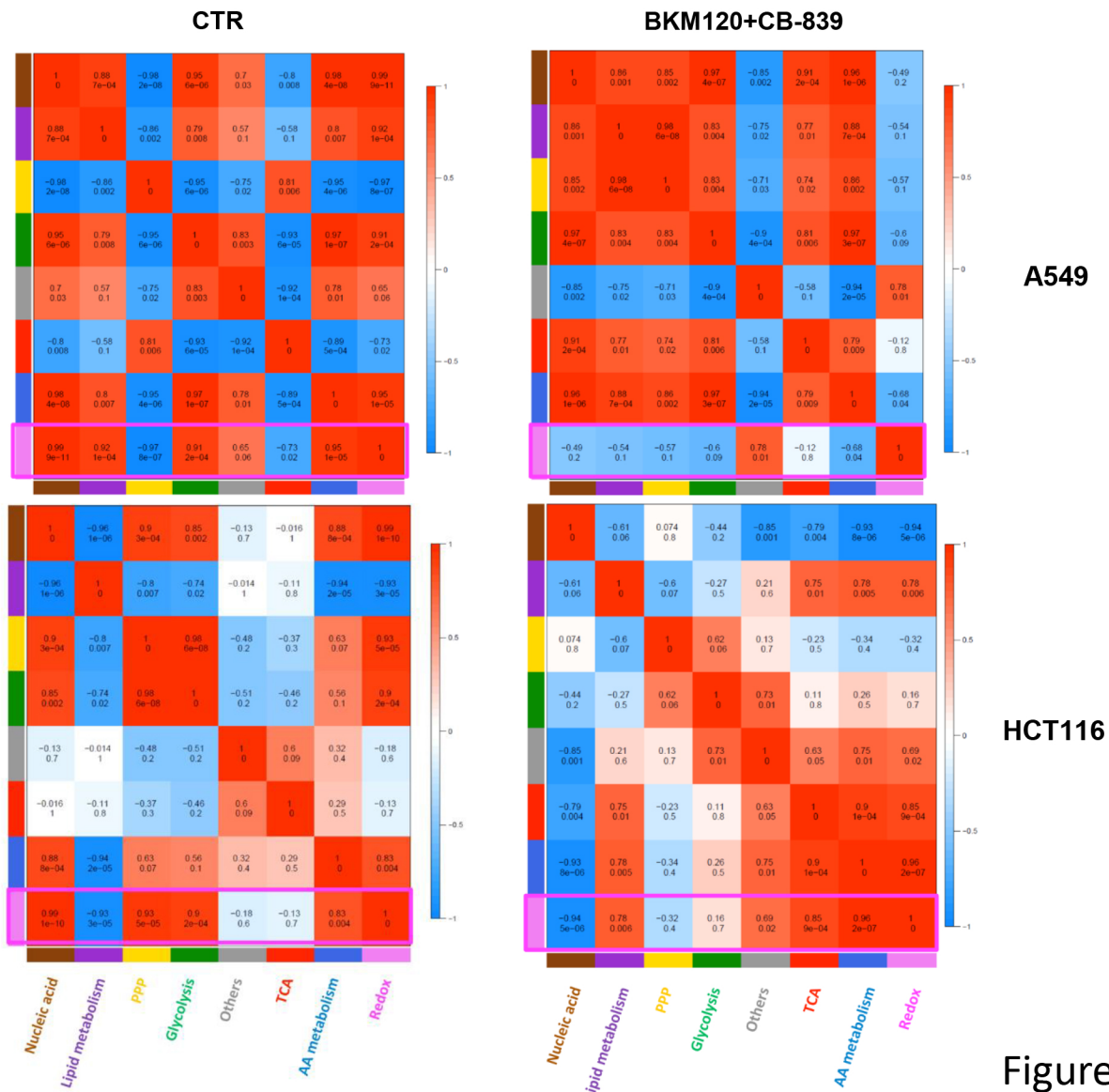**Figure S5**

Supplement: Supplementary file 1 — Additional file 1. Supplementary Materials. [file 40170_2020_227_MOESM1_ESM.zip › Figure S5.pdf]

A

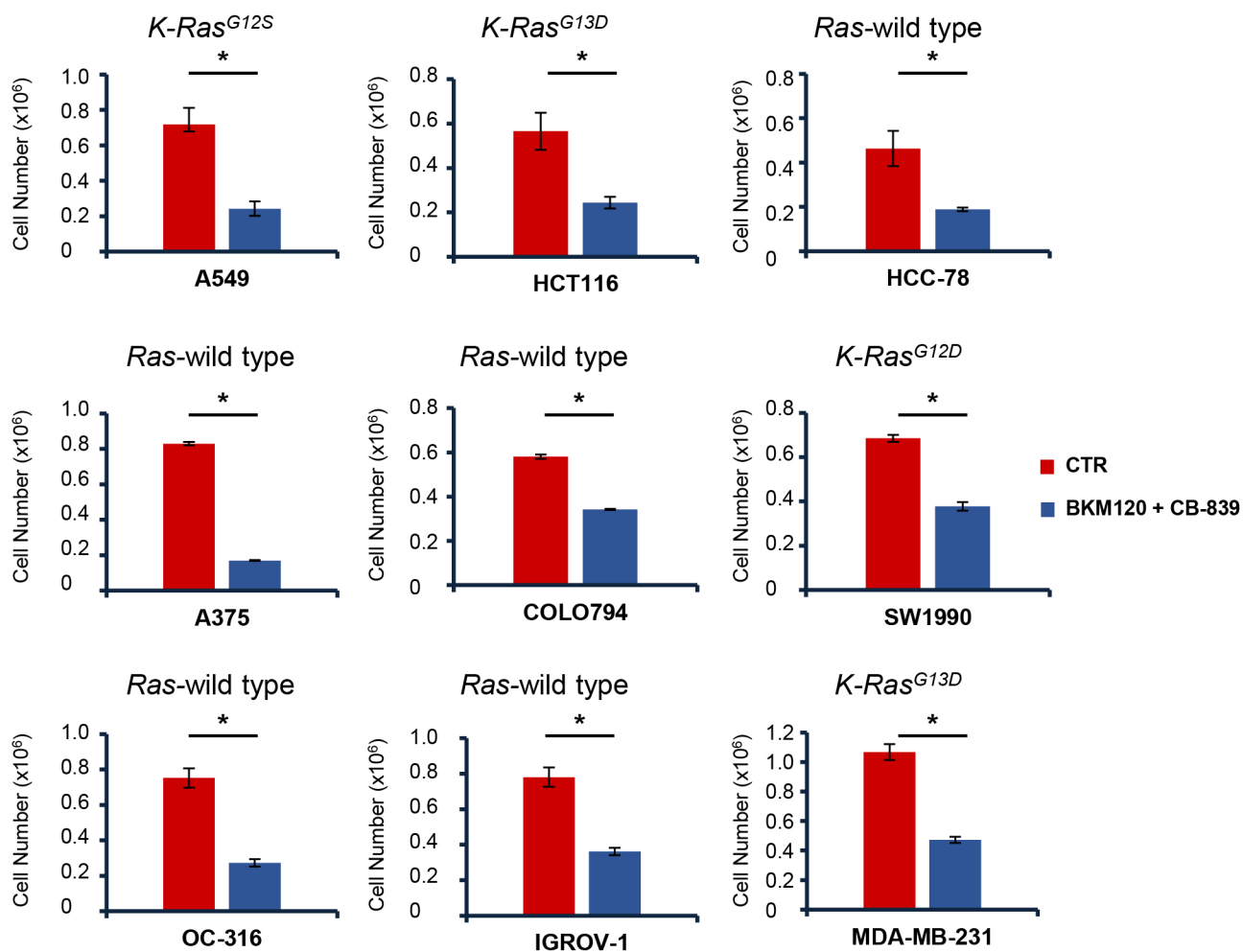

Figure S6

Supplement: Supplementary file 1 — Additional file 1. Supplementary Materials. [file 40170_2020_227_MOESM1_ESM.zip › Figure S6.pdf]
