# Supplementary material for: Disruption of redox homeostasis for combinatorial drug efficacy in K-Ras tumors as revealed by metabolic connectivity profiling
Source: Cancer Metab. 2020 Sep 29;8:22. doi: 10.1186/s40170-020-00227-4 (PMC7523077; doi:10.1186/s40170-020-00227-4)
Supplement: Supplementary file 1 — Additional file 1. Supplementary Materials. [file 40170_2020_227_MOESM1_ESM.zip › Supplementary Mat.doc]

**Supplemental information**

**Supplementary Figures Legends**

**Figure S1**

(**A**) Atom transition map of [U-^13^C_6_] glucose (blue circles) and [U-^13^C_5_] glutamine (violet circles) used to detect metabolic changes. Filled circles indicate ^13^C enrichment. Glycolysis and TCA cycle intermediates metabolites labeling from [U-^13^C_6_]Glc and [U-^13^C_5_]Gln in A549 (■) and HCT116 (■) cancer cells. **(B)** BKM120 and CB-839 dose response curves on A549 and HCT116 measured as cells per well treated for 48 hours with 0 to 20μM BKM120 or 0 to 1000nM CB-839. Error bars represent SD for n=3 biological replicates. **(C)** Aldolase activity and glutamate production in A549 and HCT116 cancer cells treated with BKM120 (1μM) or CB-839 (50nM) for 48h. (**D-E**) Post mortem analysis of animal and tumor weight of A549 (**D**) and HCT116 (**E**) tumors exposed to the combinatorial treatment compared to CTR. **(F)** Venn diagram showing the number of pairwise correlations shared between A549 and HCT116 control tissues (A), or specific for A549 and HCT116 xenografts (B and C). **(G)** Network of common connections between A549 and HCT116. In each network two metabolites are connected if their Spearman correlation is statistically significant (FDR < 0.05) and greater than a given threshold (|rho|> 0.6).

**Figure S2**

**(A-B)** Pathway enrichment analyses of A549 (A) and HCT116 (B). Enriched metabolic pathways were ranked according to their FDR values calculated by the MetPa method implemented in MetaboAnalyst 2.0 software. The pathway impact is calculated as the sum of the importance measures of the matched metabolites normalized by the sum of the importance measures of all metabolites in each pathway. **(C-D)** Relative metabolites abundances of the folate pathway in A549 (C) and HCT116 (D) cells in CTR (■) or under combined treatment (■). **(E-F)** Intracellular ROS levels in A549 (E) and HCT116 (F) in CTR or under combinatorial treatment measured by DCFDA staining. Error bars indicate SD (n=3).

**Figure S3**

**(A-B)** Atom transition map of [U-^13^C_6_] glucose (blue circles) and [U-^13^C_5_] glutamine (violet circles) used to detect metabolic changes. Filled circles indicate ^13^C enrichment. Glycolysis and TCA cycle intermediates metabolites labeling from [U-^13^C_6_]Glc and [U-^13^C_5_]Gln in A549 (A) and HCT116 (B) cancer cells in CTR or under combinatorial treatment. **(C-D)** Schematic representation and percentage isotope labeling enrichment of metabolites of alternative pathways from [U- ^13^C_6_ ]-Glc in A549 (C) and in HCT116 (D) cancer cells in CTR or under combinatorial treatment.

**Figure S4**

(**A-B**) Schematic representation of central carbon metabolism with net flux values estimated by ^13^C MFA in A549 (A) and HCT116 (B), CTR or under BKM120 plus CB-839 treatment. Arrows colors (red color= up flux, violet= down flux) and thickness represent the significantly different fluxes (based on 95% confidence intervals). (**C-D**) GSSG/GSH ratio in CTR (■) or under combined treatment (■) in A549 (C) and HCT116 (D) based on relative abundance obtained by LC-MS analysis. (**E**) Gene Ontology (GO) Biological Process terms enriched by genes found down-regulated in response to combined treatment in HCT116 colon cancer cells. Number of down-regulated differentially expressed genes (DEGs) associated to each term are shown as green bars. All the enrichments are statistically significant (blue line indicates significance expressed as -logP value) (**F**) NADP+ and NADPH levels in CTR (■) or under combined treatment (■) in A549 and HCT116 cells obtained by colorimetric assay.

**Figure S5**.

(**A-B**) Heatmaps of Spearman correlation coefficients among each pair of metabolic traits and cell number for A549 (A) and HCT116 cells (B) in control condition and under single or combined drug treatments. Rows and columns refer to metabolic traits grouped and colored according to the different classes reported in the legend. Blue or red spots refer to positive or negative correlations at a significance level of False Discovery Rate (FDR) <0.05, whereas white spots refer to not statistically significant correlations. Black boxes highlight the nucleic acid metabolism pathway under single or combined drug treatment to show loss of connectivity due to the treatment.

(**C**) A549 and HCT116 connectivity analyses in control (left panels) and under combinatorial treatment (right panels) condition. Each panel shows the heatmap of the correlation matrix between the eigenmetabolites of each pathway. Each cell of the heatmap reports the correlation value (top number) with the corresponding pvalue (bottom number) and is color-coded by correlation according to the color assignment bar displayed along the heatmap. Color changes from blue (i.e., negative correlation) to red (i.e., positive correlation). More intense shades indicate more intense inter-pathway correlations.

**Figure S6**.

Combinatorial treatment effect in several cancer cell lines. PI3K/ALDO (NVP-BKM120) inhibitor and glutaminase inhibitor (CB839) cooperate to inhibit growth of lung cancer cell lines (A549 and HCC78), colon cancer cell line (HCT116), metastatic melanoma cancer cell lines (Colo 794 and A375), triple negative breast cancer cell (MDA-MB231) and ovarian cancer cell lines (OC-316 and IGROV-1).

**Supplementary Table 1**

**Supplementary Table 2**

**Supplementary Table 3.** Table of significant up- (red boxes) or down- (green boxes) regulated metabolites involved in redox metabolism according to the KEGG database in A549 and HCT116 human cancer cells under combined drugs treatment.

**Supplementary Materials and Methods**

Cell culture

A549 and MDA-MB231 cell lines were routinely grown in Dulbecco’s modified Eagle’s medium (DMEM) containing 4mM L-glutamine, supplemented with 10% fetal bovine serum. HCT116 cell line was grown in DMEM containing 4mM L-glutamine, supplemented with 10% newborn calf serum. A375 cell line was grown in Dulbecco’s modified Eagle’s medium (DMEM) containing 2mM L-glutamine, supplemented with 10% fetal bovine serum. HCC78, Colo 794 and SW1990 cell lines were grown in RPMI containing 2mM L-glutamine, supplemented with 10% fetal bovine serum. IGROV-1 and OC-316 cell lines were grown in RPMI containing 2mM L-glutamine, supplemented with 10% fetal bovine serum, 10mM Hepes and 1mM pyruvate. All media were supplemented with 100 U/ml penicillin and 100 µg/ml streptomycin and cells were incubated at 37°C in a 5% CO_2_ incubator. All reagents for media were purchased from Life Technologies (Carlsbad, CA, USA).

Mice

Mice chosen for these studies were 7/8-weeks old nu/nu female mice (Envigo RMS S.r.l. Italy). Animals were kept under specific pathogen free conditions and experiments were carried out in compliance with institutional guidelines for the care and the use of experimental animals, which have been notified to the Italian Ministry of Health and approved by the Ethics Committee of the IRCCS San Raffaele Scientific Institute of Milan.

Cell proliferation analysis and cell treatments

Cells were plated in 6-well plates in normal growth medium. For proliferation curves under nutrient deprivation conditions, culture medium was replaced after 18h with normal growth medium, or low glutamine medium (0.5mM Gln) or low glucose medium (1 mM Glc). Cells were collected and counted after 24, 48, 72 and 144h.

For dose-response curves, cells were treated with the indicated amounts of CB-839 (MedChem Express) and BKM120 (Selleck Chemicals) for 48h and then counted. Data are expressed as percentage of survival with respect to the control condition (normal growth medium). For the proliferation curves in the presence of drugs, cells were treated with 50nM CB-839 and/or 1 µM BKM120 for 24-48-72-144h and then counted.

Metabolites quantification in the media samples

Absolute quantification of glucose, lactate, glutamine and glutamate in spent media was determined enzymatically using YSI2950 bioanalyzer (YSI Incorporated, Yellow Springs, OH, USA).

Seahorse oxygen consumption rate

Cellular oxygen consumption rate (OCR) was measured by Seahorse XF extracellular flux analyzer (Seahorse Bioscience Inc) according to the manufacturer’s instructions. Briefly, cells were seeded in Seahorse XF 24-well assay plates at a cell density of 20000 cells per well in normal growth medium. After overnight attachment, the medium was washed and replaced with pre-warmed assay medium (non-buffered DMEM supplemented with 1mM sodium pyruvate, 25mM glucose and 4mM glutamine, pH 7.4) and incubated in a non-CO_2_ incubator at 37 °C for 60 min. Basal levels of OCR were recorded followed by the mitochondrial stress test (1μM oligomycin, 1μM FCCP, 0.5μM rotenone/ antimycin A).

Aldolase activity in cell samples

Aldolase activity was measured in cell samples in the presence or absence of 1µM BKM120 using the Aldolase Activity Colorimetric Assay Kit (BioVision). Cells (6x10^5^) were homogenized in 100μl of assay buffer and then samples were processed according to the manufacturer’s protocol. Activity was read kinetically for 20 minutes at OD 450 nm using a Cary 60 UV-Vis spectrophotometer (Agilent Technologies).

ROS levels measurement

ROS levels were measured using the DCFDA Cellular ROS Detection Assay Kit (Abcam, Cambridge, UK). Cells were harvested and stained with 20μM dichloro-diyidro-fluoresceine-diacetate (DCFDA) for 30 min at 37°C. Thereafter, cells were washed and fluorescence was measured at excitation/emission wavelengths of 485nm/535nm respectively using Cary Eclipse Fluorescence Spectrophotometer (Agilent Technologies).

ATP quantification in cell samples

ATP levels were measured using ATP Colorimetric/Fluorometric Assay Kit (BioVision) according to the manufacturer’s protocol. Fluorescence was measured at excitation/emission wavelengths of 535nm/587nm respectively using Cary Eclipse Fluorescence Spectrophotometer (Agilent Technologies).

ALT and AST activity in liver tissues

Alanine Aminotransferase (ALT) Activity Fluorometric Assay Kit and Aspartate Aminotransferase (AST) Activity Colorimetric Assay Kit were purchased by Biovision. Briefly, 10 mg of mouse livers were homogenized in 200μl of appropriate assay buffer in TissueLyser II (Qiagen) for 30 seconds, 30 Hz power. Samples were then processed according to the manufacturer’s protocols. For ALT activity, fluorescence was read kinetically for 60 minutes at excitation/emission wavelengths of 535nm/587nm respectively using Cary Eclipse Fluorescence Spectrophotometer (Agilent Technologies). AST activity was assessed reading kinetically for 60 minutes at OD 450 nm using a Cary 60 UV-Vis spectrophotometer (Agilent Technologies).

NADP/NADPH assay

NADP and NADPH levels were detected using NADP/NADPH Quantitation Colorimetric Kit (BioVision Inc., Milpitas CA, USA) according to the manufacturer's protocol. Briefly, cells (1.5 × 106/well) were lysed by sonication (two cycles: 5 s for five pulses at 70% power) in 800 µl of extraction buffer. Samples (125 µl) were then directly used to detect total NADP/NADPH. To detect NADPH only, samples were heated at 60 °C for 30 min to clear out all NADP+ species. Colorimetric measurements were performed at OD 450 nm using a Cary 60 ultraviolet–visible spectrophotometer (Agilent Technologies).

Metabolites extraction from cell culture

For untargeted experiments, cells were plated in 6-well plates with normal growth medium. After 18h, cells were washed with PBS and incubated for 48h in fresh complete medium in the presence or absence of treatments. For labelling experiments, cells were incubated for 48h in fresh media supplemented with 25mM [U-^13^C_6_]-Glc or 4mM [U-^13^C_5_]-Gln or 4mM [alpha-^15^N]-Gln (purchased by Cambridge Isotope Laboratories, Inc.) in the presence or absence of treatments.

Metabolites extraction for GC-MS analysis was performed as described previously [13]. Briefly, cells were quenched with 1:1 ice-cold methanol:water and collected by scraping. After sonication, one volume of chloroform was added, and cells were vortexed at 4°C for 20 min. Samples were centrifuged at 12000g for 10 min, and the aqueous phase was collected in a new tube and evaporated under airflow at 37°C.

For metabolites extraction for LC-MS analysis, cells were quickly rinsed with NaCl 0.9% and quenched with 500 µl ice-cold 70:30 acetonitrile-water. Plates were placed at -80°C for 10 minutes, then the cells were collected by scraping. Cells were sonicated 5 sec for 5 pulses at 70% power twice. Samples were centrifuged at 12000g for 10 min and the supernatant was collected in a new tube and evaporated under air flow at 37°C. Samples were then resuspended with 150 μl of H_2_O prior to analyses.

Metabolites extraction from tissue samples

Metabolites extraction for GC-MS analysis was performed as described previously [13]. Briefly, 0.1 ml ice-cold methanol was added to 10mg of tissue. An equal volume of water was added and samples were sonicated, incubated at -80°C and sonicated again. One volume of chloroform was added and samples were vortexed at 4°C for 30 min and then centrifuged at 12000g for 10 min. The aqueous phase was recovered and evaporated under airflow at 37°C.

For metabolites extraction for LC-MS analysis, the protocol was adapted from Naz et al [15]. Briefly, 100 µl ice-cold 50:50 methanol-water was added to 10mg of tissue and samples were sonicated 5 sec for 5 pulses at 70% power twice. After a 30 min incubation at -80°C, samples were sonicated as described above. A total of 100μL of homogenate was vortexed with 320μL of ice-cold methanol for 2 min. Then, 80μL of methyl-tert-butyl ether (MTBE) was added and samples were placed on a shaker for 1h at room temperature. Extracted samples were then centrifuged at 12000g for 20 min. 400μL of supernatant was recovered and evaporated under air flow at 37°C. Samples were resuspended with 150μl of H_2_O prior to analyses.

GC-MS metabolic profiling

Derivatization was performed using automated sample prep WorkBench instrument (Agilent Technologies). Dried polar metabolites were dissolved in 60μl of 2% methoxyamine hydrochloride in pyridine (Pierce) and held at 40°C for 6h. After the reaction, 90μl of MSTFA (N-Methyl-N-(trimethylsilyl) trifluoroacetamid) was added and samples were incubated at 60°C for 1h. Derivatized samples were analyzed by GC-MS using a DB-35MS column (30 m x 0.25mm i.d. x 0.25 µm) installed in an Agilent 7890B gas chromatograph (GC) interfaced with an Agilent 7200 Accurate-Mass Quadrupole Time-of-Flight (QTOF) mass spectrometer (MS) operating under electron impact (EI) ionization at 70eV. Samples (1μl) were injected in a splitless mode at 250°C, using helium as the carrier gas at a flow rate of 1 ml/min. The GC oven temperature was held at 100°C for 2 min and increased to 325°C at 10°C/min. GC/MS data processing was performed using Agilent Muss Hunter software and statistical and pathway analyses were performed using Mass Profiler Professional (MPP) software and MetaboAnalyst 4.0 [15]. Relative metabolites abundance was carried out after normalization to internal standard d27 Myristic acid and cell number.

For labeling experiments, dried polar metabolites were dissolved in 60μl of 2% methoxyamine hydrochloride in pyridine (Pierce) and held at 40°C for 6h. After the reaction, 90μl of MTBSTFA + 1% TBDMCS (Pierce) were added and samples were incubated at 60°C for 1h. Sample (1μl) were injected in splitless mode at 270°C, using helium as the carrier gas at a flow rate of 1 ml/min. The GC oven temperature was held at 100°C for 3 min and increased to 300°C at 3.5°C/min. Data were pre-processed using the OpenChrom software package [16]. Raw intensity values across accurate masses were first binned into unit masses (i.e. 50, 51, 52, etc. ± 0.1 m/z) and exported as “.csv” files followed by conversion to NetCDF using the OpenChrom software’s file converters. Mass isotopologue distributions (MIDs) were determined using Matlab by integrating metabolite ion fragments and correcting for natural abundance using in-house algorithms adapted from [17].

LC-MS metabolic profiling

LC separation was performed using an Agilent 1290 Infinity UHPLC system and an InfintyLab Poroshell 120 PFP column (2.1 x 100 mm, 2.7 μm; Agilent Technologies). Mobile phase A was water with 0.1% formic acid. Mobile phase B was acetonitrile with 0.1% formic acid. The injection volume was 15 μL and LC gradient conditions were: 0 min: 100% A; 2 min: 100% A; 4 min: 99% A; 10 min: 98% A;11 min: 70% A; 15 min: 70% A; 16 min: 100% A with 5 min of post-run. Flow rate was 0.2 mL/min and column temperature was 35°C. MS detection was performed using an Agilent 6550 iFunnel Q-TOF mass spectrometer with Dual JetStream source operating in negative ionization mode. MS parameters were: gas temp: 285°C; gas flow: 14 l/min; nebulizer pressure: 45psig; sheath gas temp: 330°C; sheath gas flow: 12 l/min; VCap: 3700 V; Fragmentor: 175 V; Skimmer: 65 V; Octopole RF: 750 V. Active reference mass correction was through a second nebulizer using masses with m/z: 112.9855 and 1033.9881. Data were acquired from m/z 60–1050. Data analysis and isotopic natural abundance correction was performed with MassHunter ProFinder and MassHunter VistaFlux software (Agilent).

Animal model and pharmacological therapy

A549 *k-ras* ^G12S^ (5 x 10^6^) or HCT116 *k-ras* ^13D^ (2 x 10^6^) cells in 200 ul of phosphate-buffered saline (PBS) and Matrigel mixed at 1:1 were injected subcutaneously (s.c) on the right flank of 7/8-weeks old nu/nu female mice (Envigo RMS S.r.l. Italy). After cells injection, mice were monitored twice a week for body weight. Tumor volume was measured using a digital caliper and calculated following the formula: tumor volume = (long side * (short side)^2^) / 2.

NVP-BKM120 was formulated in NMP/PEG300 (10/90, v/v). Solution was freshly prepared daily just before gavaging by dissolving the powder, first in N-Methyl-2-pyrrolidone (NMP, Sigma Aldrich) with sonication and then by adding the remaining volume of PEG300 (Sigma Aldrich) as previously described [19]. The application volume was 10 mL/kg. CB-839 was dissolved in a vehicle containing 25% (w/v) hydroxypropyl-b-cyclodextrin (Cayman Chemical Company) in 10mmol/L citrate (pH 2.0). The formulation was 20 mg/mL for a final dosing volume of 10 mL/kg as previously described [12]. When a mean tumor volume reached 130-150 mm3, mice were divided into groups (n = 7-12) and orally administered with vehicles (CTR) or with a combination of CB-839 (200 mg/kg twice daily, 5 d per week) and NVP-BKM120 (50 mg/kg daily, 5 d per week) for two weeks. At the end of the study, animals were sacrificed and tumors collected for post mortem metabolomics analyses. For labelling experiments, 1M [U-13C6] glucose in sterile PBS was infused injecting 80 μl (20 mg) of solution at 15 min intervals 3 times before the sacrifice. For survival study, treatments were administered until either a tumor dimension reached 15 mm or both dimensions exceeded 10 mm or for evident signs of disease (i.e. motor difficulty). To calculate Tumor Growth Inhibition, we used the following formula: TGI = [1 - (TF/T0)A/(TF/T0)V] X 100, where TF is the time point analyzed, T0 is the initial time, A is the corresponding drug, and V is the vehicle [20].

Positron Emission Tomography imaging with [^18^F] Fluorodeoxyglucose and quantification

PET images were obtained using a YAP-(S)-PET II small animal scanner (ISE s.r.l., Pisa, Italy). Animals were acquired before therapy, after 7 days (1 week) and after 14 days (2 weeks) from the beginning of therapy. Acquisitions and images analysis and quantification were performed as previously described 13]..

PET imaging and quantification

PET images were obtained using a YAP-(S)-PET II small animal scanner (ISE s.r.l., Pisa, Italy). Animals were acquired before therapy, after 7 days and after 15 days. Acquisitions and images analysis and quantification were performed as previously described [13].

Transcriptome sequencing (RNA-seq) experiments and data analysis

Total RNA was extracted from A549 lung cancer and HCT116 colon cancer cell lines before and after combined treatment, checked for integrity by microcapillary electrophoresis on 2200 TapeStation instrument (Agilent Technologies, Santa Clara, CA, USA) and stored at -80° until use. RNA-seq libraries were prepared using the Illumina TruSeq Stranded mRNA Library Prep Kit (Illumina, San Diego, CA, USA), according to manufacturers’ instructions, and sequenced on HiSeq2500 platform (Illumina) in a 150-cycle paired-end run at Biodiversa Srl (Trento, Italy). Three independent replicates were sequenced for each condition (untreated and treated) for both cell lines. Raw reads are available in NCBI Short Reads Archive (SRA) under Accession Number PRJNA623750.

After fastq quality control by using FastQC tool, raw reads were trimmed to 100bp and mapped to the human reference genome (hg38) using STAR aligner (v.2.6.1d). Gene counts were calculated by HTSeq (v.0.6.1), using the hg38 Encode-Gencode GTF file (v28) as gene annotation file. Differential gene expression analysis of treated versus untreated cell lines was carried out using DESeq2 software (v.1.24.0). Low expressed genes (sum of read counts across all samples < 1), as well as outliers between replicates, were filtered out before testing genes for statistical significance. A |log2FC| > 1 and adjusted p-value (padj, Benjamini-Hochberg (BH) correction) < 0.1 were used as cut-off to define statistically significant differentially expressed genes (DEGs). ToppGene suite was used to perform gene enrichment analysis for Gene Ontology (GO) categories and biological pathways (KEGG, Biocarta, Reactome) (https://toppgene.cchmc.org/). A FDR (BH correction) < 0.1 was applied to all the annotation terms to defined statistically significant enrichment.
